# Casein Kinase 1 and Phosphorylation of Cohesin Subunit Rec11 (SA3) Promote Meiotic Recombination through Linear Element Formation

**DOI:** 10.1371/journal.pgen.1005225

**Published:** 2015-05-20

**Authors:** Naina Phadnis, Lubos Cipak, Silvia Polakova, Randy W. Hyppa, Ingrid Cipakova, Dorothea Anrather, Lucia Karvaiova, Karl Mechtler, Gerald R. Smith, Juraj Gregan

**Affiliations:** 1 Division of Basic Sciences, Fred Hutchinson Cancer Research Center, Seattle, Washington, United States of America; 2 Max F. Perutz Laboratories, University of Vienna, Vienna, Austria; 3 Cancer Research Institute, Slovak Academy of Sciences, Bratislava, Slovakia; 4 Department of Genetics, Comenius University, Bratislava, Slovakia; 5 Research Institute of Molecular Pathology, Vienna, Austria; The University of North Carolina at Chapel Hill, UNITED STATES

## Abstract

Proper meiotic chromosome segregation, essential for sexual reproduction, requires timely formation and removal of sister chromatid cohesion and crossing-over between homologs. Early in meiosis cohesins hold sisters together and also promote formation of DNA double-strand breaks, obligate precursors to crossovers. Later, cohesin cleavage allows chromosome segregation. We show that in fission yeast redundant casein kinase 1 homologs, Hhp1 and Hhp2, previously shown to regulate segregation via phosphorylation of the Rec8 cohesin subunit, are also required for high-level meiotic DNA breakage and recombination. Unexpectedly, these kinases also mediate phosphorylation of a different meiosis-specific cohesin subunit Rec11. This phosphorylation in turn leads to loading of linear element proteins Rec10 and Rec27, related to synaptonemal complex proteins of other species, and thereby promotes DNA breakage and recombination. Our results provide novel insights into the regulation of chromosomal features required for crossing-over and successful reproduction. The mammalian functional homolog of Rec11 (STAG3) is also phosphorylated during meiosis and appears to be required for fertility, indicating wide conservation of the meiotic events reported here.

## Author Summary

The formation of haploid gametes (sex cells, such as eggs and sperm) from diploid precursor cells involves two nuclear divisions but one round of chromosomal replication. In the unique first meiotic division, centromeres of sister chromatids remain connected and homologous chromosomes (homologs) segregate from each other. In most species proper homolog segregation requires that crossover recombination occur between homologs to impart tension between homologs as they move apart. A protein kinase (casein kinase 1) has long been known to regulate proper sister centromere connections by phosphorylating Rec8, a meiosis-specific sister chromatid cohesin subunit. We report here that in fission yeast this kinase has a second critical role—to mediate phosphorylation of another meiosis-specific cohesin subunit Rec11. Phosphorylation of Rec11 enhances loading of two meiosis-specific components of linear elements, which are related to the synaptonemal complex and help pair homologs. These linear element proteins lead to high-level DNA breakage and crossovers between homologs. Thus, casein kinase regulates two crucial but separate events in meiosis. The mammalian functional homolog of Rec11, called STAG3, is also phosphorylated during meiosis and appears to be required for fertility in human females. These observations suggest wide-spread conservation of the roles of casein kinase 1 and Rec11 in ensuring proper meiotic chromosome segregation and sexual reproduction.

## Introduction

The two specialized nuclear divisions during meiosis convert a diploid precursor cell into one or more haploid cells (gametes). Uniquely in the first meiotic division, centromeres of homologous chromosomes (homologs) segregate from each other, whereas centromeres of sister chromatids segregate from each other only in the second meiotic division, as in mitotic divisions. Proper chromosome segregation is essential for the formation of gametes with viable chromosome complements and requires two chromosomal events special to meiosis—crossing-over between homologs, and spatially and temporally regulated cohesion between sister chromatids. For homologs to segregate properly from each other in the first meiotic division, they must pair and the sister centromeres must remain connected and move as a unit to one pole of the cell; in most species pairing is accompanied by physical exchange of DNA between homologs to form crossovers that provide the interhomolog tension required for their proper segregation. For sister centromeres to segregate properly from each other in the second meiotic division, pericentric cohesion must be established in the first division but be removed only during the second division. This is accomplished by protection of a meiosis-specific cohesin subunit only at and near the centromere before and during the first division and its degradation specifically just before the second division.

Although the outlines of these two events are known [1], how they are regulated and coordinated remains unclear. Previous data and our new results reported here show that casein kinase 1 plays an essential role in both of these events, by mediating the phosphorylation of two separate subunits of cohesin. Our observations are in fission yeast, but the wide-spread conservation of these subunits suggests that our conclusions apply broadly to eukaryotes.

Meiotic cohesin is a large protein complex composed of Smc1 and Smc3, which are common to both the mitotic and meiotic forms, and the meiosis-specific Rec8 and Rec11 subunits [2–5]. (Some species, including budding yeast and Tetrahymena, lack a clear Rec11 homolog and retain the mitotic form, Scc3, in meiosis [6,7].) This complex forms a ring that connects sister chromatids from the time of replication to the time of chromatid segregation [8,9]. To allow segregation, Rec8 is phosphorylated and then cleaved by a protease called separase [10]. During meiosis, Rec8 cleavage occurs in two steps: along the chromosome arms during the first meiotic division (MI) and in the pericentric region during the second meiotic division (MII) [11]. In MI pericentric Rec8 is protected by shugoshin (Sgo1), which recruits PP2A protein phosphatase and thereby prevents Rec8 cleavage. During MII, cohesin is no longer protected from separase, which then cleaves pericentric Rec8 to allow sister centromere segregation [12]. In the absence of Rec8, chromosome segregation is like that in mitosis: sister chromatids, rather than homologs, segregate at MI [13]. In the absence of Rec11, chromosome segregation is similar to that in a recombination-deficient mutant: sister centromeres remain connected until MII, when they segregate, but aberrant arm cohesion and a paucity of crossovers reduce proper homolog segregation at MI [5].

Rec8 and Rec11 are also required for formation of crossovers, which result from the repair of DNA double-strand breaks (DSBs) programmed to occur during meiosis [14,15]. DSBs are made by the highly conserved topoisomerase-like protein Spo11 (named Rec12 in fission yeast) [16]. To be active, Rec12 requires six essential partner proteins, which likely function as a large complex similar to that of the Spo11 complex of budding yeast [17]. In a proposed pathway, Rec8 and Rec11 cohesin subunits are loaded onto chromosomes during S phase [14,18]. Their loading allows loading of the linear element (LinE) complex, related to the synaptonemal complex of other species; LinEs contain Rec10, Rec25, Rec27, and Mug20 [14,19–21]. In accord with this pathway, LinEs are rare or absent in each of the six corresponding mutants [14,19–22]. LinE protein loading activates the Rec12 complex to make DSBs [14]. Although Rec10 is required for DSB-formation and recombination throughout the genome, the other three LinE proteins, like Rec8 and Rec11, are required more strongly in some chromosomal intervals than in others [14].

Cleavage of Rec8 to allow proper chromosome segregation requires phosphorylation of Rec8 by one or more protein kinases, including casein kinase 1 orthologs in both budding and fission yeasts [23]. In fission yeast two casein kinase 1 paralogs, Hhp1 and Hhp2 (collectively called Hhp here), function redundantly to phosphorylate Rec8 [12,23]. In their absence Rec8 cleavage is delayed and persistent sister-chromatid cohesion along chromosome arms often prevents chromosome segregation, leading to many fewer viable gametes (spores) than in wild-type cells. Because of the close connection between meiotic chromosome segregation and recombination, exemplified by the role of Rec8 in both processes [13,15], we examined meiotic recombination in Hhp-deficient mutants. We found that Hhp is indeed required for recombination but that the substrate for this process is, unexpectedly, the meiosis-specific cohesin subunit Rec11, not Rec8. Our findings indicate that Hhp regulates chromosome segregation and recombination separately, by regulating Rec8 cleavage and by activating Rec11 to promote DSB-formation and recombination. We discuss parallels in the roles of meiotic cohesin subunits common to fission yeast and mammals.

## Results

### Casein kinase 1 (*hhp*) null mutants are meiotic recombination-deficient

To test for a possible role of casein kinase 1 homologs Hhp1 and Hhp2 in meiotic recombination of the fission yeast *Schizosaccharomyces pombe*, we measured recombination in *hhp1Δ hhp2Δ* double deletion mutants. Recombination was reduced by factors ranging from about 5 to 170, depending on the interval measured (Table 1). Both intergenic recombination (crossing over) and intragenic recombination (gene conversion) were reduced in the double mutant but only slightly in each single mutant, indicating that Hhp1 and Hhp2 have redundant roles in recombination, as previously reported for chromosome segregation [12]. Similar differential reductions, depending on the interval measured, are observed in cohesin- and LinE-deficient mutants [19,24,25], leading us to suspect that the Hhp substrate required for recombination is a cohesin subunit or LinE protein. Our results below bear out this suspicion.

**Table 1:** Meiotic recombination depends on redundant Hhp1 and Hhp2 kinases.

		Genetic distance		Fold reduction^d^	Genetic distance	
Genetic interval	Chr	*hhp1* ^*+*^ *hhp2* ^*+*^	*hhp1∆ hhp2∆*		*hhp1∆*	*hhp2∆*
*arg3A —tps16* ^a^	3	21 ± 2.1	1.7 ± 0.5	12	– ^e^	– ^e^
*mat1—ade1* ^a^	2	98 ± 2.1 ^c^	23 ± 0.8	4.9	86 ± 2	105 ± 22
*ade1—lys4* ^a^	2	50 ± 2.9	7.3 ± 1.0	6.8	45 ± 3	42 ± 0.4
*ade6-M26—ade6-469* ^b^	3	7160 ± 460	42 ± 14	170	3550 ± 720	6500 ± 370
*ade6-M375—ade6-469* ^b^	3	360 ± 34 ^c^	6.3 ± 1	57	77 ± 6	480 ± 70

^a^ Data are cM, calculated from the observed recombinant fraction using Haldane’s equation.

^b^ Data are the mean ± SEM of Ade^+^ recombinants per million viable spores.

^c^ Data are from [62].

^d^ Ratio of genetic distance in wt to that in *hhp1∆ hhp2∆* mutant.

^e^ Not determined.

### An ATP analog-sensitive *hhp* mutant is DSB- and recombination-deficient even without analog

Because mitotic growth and viable spore yield are severely impaired in *hhp1Δ hhp2Δ* mutants [26,27], we used an Hhp1 mutant (*hhp1-as* encoding the Met^84^ → Gly alteration) sensitive to 1-NM-PP1, an analog of the purine moiety of ATP, by alteration of its ATP-binding site [28,29]. In conjunction with *hhp2Δ* we could thereby allow Hhp function (in the absence of analog) or block Hhp function (in its presence). As expected from the results above, recombination was strongly reduced in the presence of the analog: we observed ~15- and 100-fold reductions in the two intervals tested (Table 2), comparable to the reductions seen in *hhp1Δ hhp2Δ*. Recombination was also reduced to essentially the same extent in the *absence* of the analog, a condition that allowed much higher viable spore yield and nearly wild-type chromosome segregation (Table 2 and S1 Table). This fortuitous result, presumably a reflection of *hhp1-as* allowing adequate phosphorylation of some substrates but not others, allowed us to conduct experiments under conditions allowing nearly wild-type mitotic growth and high viable spore yield. We discuss later the putative separation of functions of *hhp1-as*.

**Table 2:** Even without ATP-analog, *hhp1-as hhp2∆* mutant has strongly reduced meiotic recombination but only weakly reduced viable spore yield.

Relevant genotype	*ade6—arg1* ^a^		*ade6-M26* x *ade6-52* ^b^		Relative viable spore yield ^c^	
	*–* analog	+ analog	*–* analog	+ analog	*–* analog	+ analog
*hhp1* ^*+*^ *hhp2* ^*+*^	64	71	3800	1300	100 (2)	35 (2)
*hhp1-as hhp2∆*	2	5	9	12	29 (4)	0.08 (4)
*rec12∆*	<0.7 ^d^	– ^e^	<5 ^f^	–	22 (2)	14 (2)
*hhp1-as hhp2∆ rec12∆*	–	–	–	–	35 (4)	0.14 (4)

^a^ Data are cM, calculated from the observed recombinant fraction using Haldane’s equation.

^b^ Data are the number of Ade^+^ recombinants per million viable spores.

^c^ Data, from (n) crosses, are the mean viable spore yield per viable cell of the less numerous parent in each cross relative to that of hhp1^+^ hhp2^+^ without analog (4.9).

^d^ Data are from [63].

^e^ Not determined.

^f^ Data are from [64].

When we coupled the *hhp1-as hhp2Δ* mutations with *rec12Δ*, we obtained results indicating that, in the absence of analog, recombination but not Rec8 cleavage was defective in the *hhp1-as hhp2Δ* mutant. Because *S*. *pombe* has only three chromosomes and has a mechanism that enhances proper segregation of non-recombinant homologs [30], *rec12Δ* mutants form ~25% as many viable spores as wild type (Table 2) [31]. This yield was not further reduced by the *hhp1-as hhp2Δ* mutations in the absence of analog, but it was greatly reduced in its presence, as expected from failure of Rec8 to be cleaved under this condition [12,32]. In the absence of analog, the failure of *rec12Δ* to further reduce the viable spore yield of *hhp1-as hhp2Δ* indicates that without analog *hhp1-as hhp2Δ* blocks recombination but not chromosome segregation. We infer that one or more recombination-promoting protein(s) is not properly phosphorylated by Hhp1-as in the absence of the analog and that in the presence of the analog Rec8 is also hypo-phosphorylated.

Meiotic recombination requires both formation and repair of DSBs. *hhp1Δ hhp2Δ* mutants have DNA repair defects in vegetative cells [26]. To determine if meiotic DSB repair is blocked in the *hhp1-as hhp2Δ* mutant in the absence of the analog, we artificially introduced DSBs with the I-*Sce*I homing endonuclease, controlled by the meiosis-specific *rec12* promoter [33]. DSBs were introduced in the *ade6* gene at the site of the *ade6-3061* mutation, which can recombine with another mutation *ade6-52* to generate Ade^+^ recombinants. In the absence of analog the frequency of recombinants was indistinguishable in wild type and in *hhp1-as hhp2Δ*, indicating that under this condition Hhp is not required for DSB repair (S2 Table). *rec8Δ*, however, reduced the recombinant frequency by a factor of ~3, suggesting that Rec8 is required for DSB repair of I-*Sce*I DSBs, as it is at some chromosomal sites in budding yeast [6]. We infer that during meiosis Hhp is required for DSB formation, but we found no evidence for its having a role in DSB repair under this condition. This conclusion is consistent with the *hhp* mutant having high viable spore yield but low recombination-proficiency in the absence of analog (Table 2).

**Fig 1:**
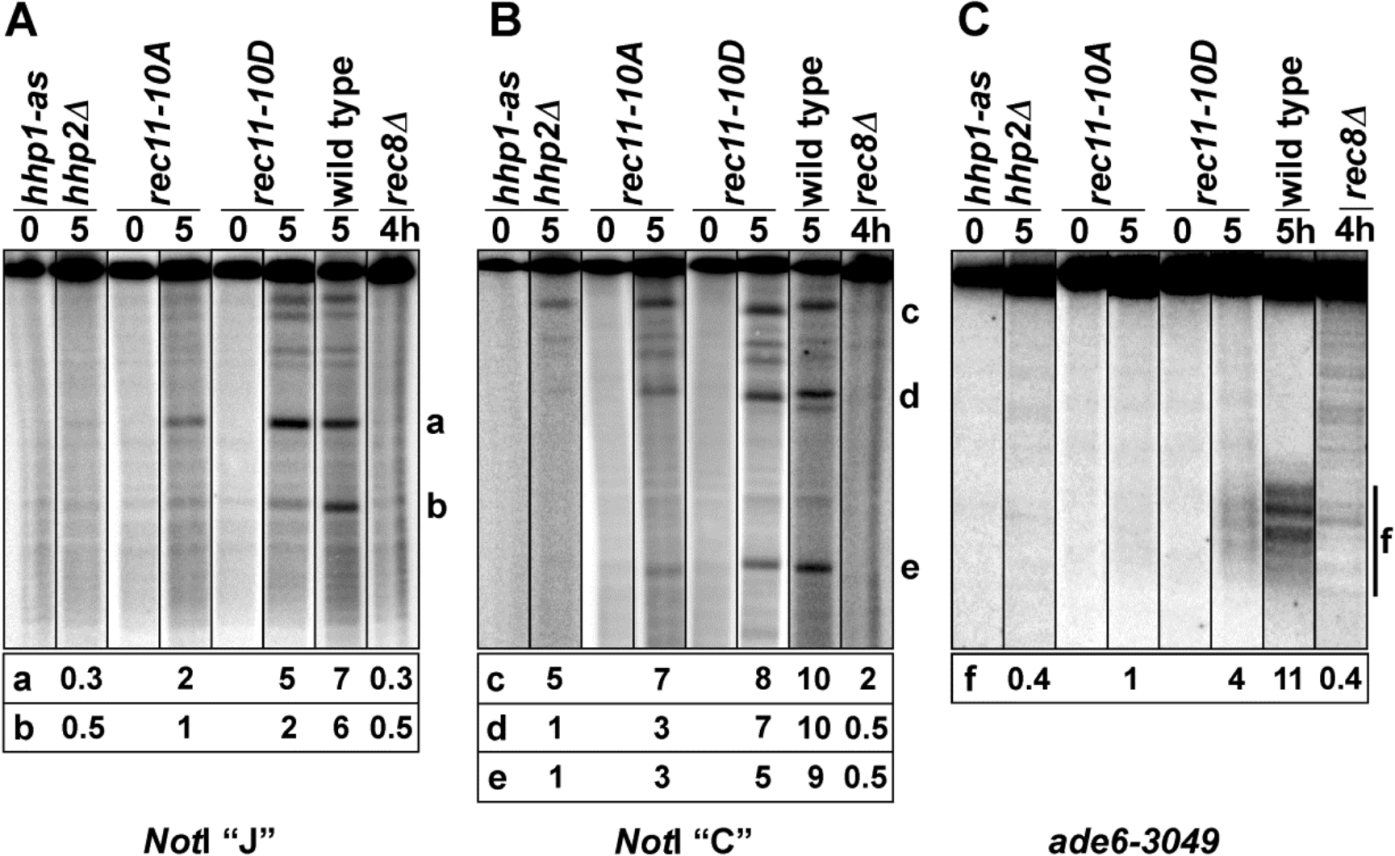
Meiotic DNA breakage in *hhp*, *rec11*, and *rec8* mutants. Strains with the indicated mutations were induced for meiosis in the absence of an ATP analog. At the indicated times, DNA was extracted and analyzed by Southern blot hybridization. All time points for each mutant were run on the same gel, one gel for the two *Not*I fragments and another for the *ade6-3049* fragment (S1 Fig). Data below each lane with meiotically induced DNA are the percent of total DNA in the bands labeled a—f after subtraction of the intensity in the corresponding 0 hr lane. These data reflect DNA breakage at DSB hotspots. DSBs at the *ade6-3049* hotspot are spread over about 1 kb, indicated by the bar (f) on the right. See also S1 Fig. (A) The 501 kb *Not*I fragment J on chromosome 1 was analyzed with a probe at its left end [60]. (B) The 1.5 Mb *Not*I fragment C on chromosome 2 was analyzed with a probe near its left end [18]. (C) The 6.6 kb AflII fragment containing *ade6* on chromosome 3 was analyzed with a probe at its right end [61].

To directly test for a role of Hhp in DSB formation, we assayed DSBs by Southern blot hybridizations of DNA extracted from *hhp1-as hhp2Δ* mutants induced for meiosis in the absence of analog; DNA from wild type and *rec8Δ* was analyzed for comparison (Fig 1 and S1 Fig). In wild type there were six prominent, meiosis-dependent DSB hotspots on the 501 kb *Not*I restriction fragment J, as seen before [34,35]. DSBs were barely detectable at these sites in *hhp1-as hhp2Δ*, as is the case in *rec8Δ* and *rec11Δ* [14,18]. DSBs were also barely detectable in *hhp1-as hhp2Δ* at the strong DSB hotspot *ade6-3049*, as is the case in *rec8Δ* and *rec11Δ* [14,18]. In contrast, DSBs were detectable, though at reduced levels, at some hotspots on the 1500 kb *Not*I restriction fragment C in *hhp1-as hhp2Δ* and in *rec8Δ* and *rec11Δ* (Fig 1B) [14,18]. These results show that Hhp is required for most meiotic DSB formation, but residual DSBs with a spatial pattern similar to that in *rec8Δ* and *rec11Δ*, which are indistinguishable [14,18], remain in the absence of Hhp function.

### Rec8 phosphorylation-deficient mutants are not recombination-deficient

Because the residual patterns of DSB formation and recombination in *hhp1-as hhp2Δ* resemble those in *rec8Δ* (Fig 1) [14,18] and because Rec8 is an Hhp substrate [12,23], we tested the hypothesis that the Hhp substrate required for DSB formation is Rec8. One of the Hhp-dependent phosphorylation sites on Rec8 (S412) is critical for cleavage of Rec8 to allow chromosome segregation [12]. The non-phosphorylatable mutant *rec8-S412A* was, however, as recombination-proficient as wild type (S3 Table) and had DSB patterns on *Not*I fragments J and D similar to those of wild type. Similar recombination results were obtained with six additional *rec8* mutants lacking seven, 12, 13, 17, or 18 phosphorylation sites (S3 Table). In *S*. *cerevisiae*, Rec8 phosphorylation is also not essential for meiotic recombination, although crossing-over is about one-half as frequent and delayed relative to wild type [36]. These results suggest that Rec8 is not, under the conditions used here, the major substrate of Hhp required for DSB formation and recombination, although minor effects cannot be ruled out.

### Rec11 is phosphorylated in an Hhp-dependent manner

To search for additional Hhp substrates during meiosis, we immunoprecipitated TAP-tagged versions of Hhp1 and Hhp2 and analyzed the precipitated proteins by mass spectrometry (S2 Fig, S4 Table and S5 Table). In meiotically induced cells, but not in mitotically growing cells, we found that Hhp1 co-immunoprecipitated with Hhp2-TAP and, conversely, Hhp2 with Hhp1-TAP. We confirmed this interaction in meiotic extracts by standard co-immunoprecipitation and Western blotting (S3 Fig). Physical interaction between Hhp1 and Hhp2 was previously observed in checkpoint-activated mitotic cells [37]. Importantly, we found the cohesin subunits Rec8, Psm1, and Psm3 (orthologs of Smc1 and Smc3 in other species) in precipitates of Hhp1 and Hhp2 from meiotic cells (S4 Table). To test the possibility that other cohesin subunits interact with Hhp, we analyzed Rec11-TAP precipitates, in which we found known cohesin subunits and Hhp2, suggesting that cohesin and Hhp physically interact. Interestingly, we did not detect interaction between Hhp and cohesin in extracts from mitotically growing cells, suggesting that this interaction is stronger during meiosis or is meiosis-specific (S4 Table).

**Fig 2:**
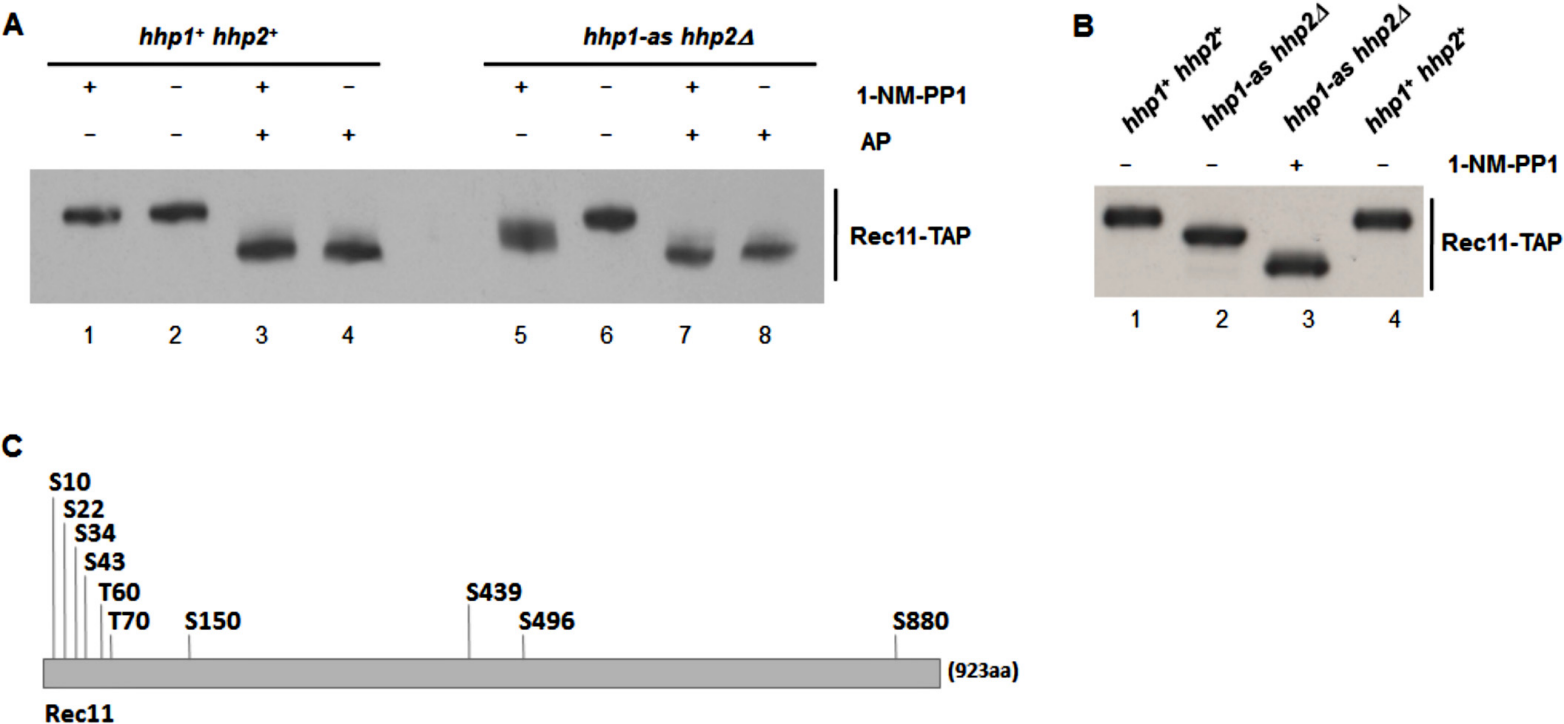
Rec11 is phosphorylated during meiosis, at least partially dependent on Hhp. (A and B) *pat1-114 rec11-TAP* cells (*hhp1* ^*+*^ *hhp2* ^*+*^ or *hhp1-as hhp2Δ*, as indicated) were induced for meiosis in medium containing (+) or lacking (–) 30 μM 1-NM-PP1, an ATP analog. Extracted proteins were treated with alkaline phosphatase (AP) (+) or not (–) and separated by gel electrophoresis. Rec11-TAP protein was detected by western blotting using peroxidase anti-peroxidase antibody. (C) Positions of phosphorylation on Rec11-TAP isolated from meiotically induced *pat1-114* cells were determined by mass spectrometry; S, serine; T, threonine. Their codons were mutated to create *rec11-10A* (encoding alanine at each position) and *rec11-10D* (encoding aspartate at each position).

To test directly for phosphorylation of Rec11, we determined the mobility of Rec11-TAP by gel electrophoresis (Fig 2). The mobility of Rec11 from wild-type cells was considerably increased by phosphatase treatment (Fig 2A, lanes 1 and 2 *vs*. lanes 3 and 4), indicating that Rec11 indeed was phosphorylated during meiosis. In the presence of analog the mobility of Rec11 from the *hhp1-as hhp2Δ* mutant induced into meiosis was greater than that from wild-type cells (lane 5 *vs*. lane 1); its mobility was further increased by phosphatase treatment (lanes 7 and 8 *vs*. lanes 5 and 6). As expected, the mobility of Rec11 from wild type (*hhp* ^*+*^) was the same with or without analog (lane 1 *vs*. lane 2), demonstrating that the increased mobility of Rec11 from the mutant was due to inhibition of Hhp and not to an off-target effect. In the absence of analog the mobility of Rec11 from the *hhp1* mutant was also greater than that from wild type (Fig 2B, lanes 1 and 4 *vs*. lane 2) and was further increased by adding analog (Fig 2B, lane 2 *vs*. lane 3), as also evident in Fig 2A (lane 5 *vs*. lane 6).

These results indicate that Rec11 phosphorylation in the *hhp1-as hhp2Δ* mutant is reduced even in the absence of the inhibitor, consistent with the *hhp1-as hhp2Δ* mutant having a recombination-deficient phenotype in the absence of inhibitor (Table 2). They also indicate that Rec11 phosphorylation is reduced even more, but not completely, in the presence of analog. This outcome is consistent with viable spore yield being somewhat reduced in the absence of analog but reduced much more in the presence of analog (Table 2). Residual Rec11 phosphorylation in the presence of analog (Fig 2A, lane 5 *vs*. lane 7) may depend on residual Hhp1-as function or on another protein kinase.

### Rec11 phosphorylation-site mutants are recombination- and DSB-deficient but segregation-proficient

To determine the nature of Rec11 phosphorylation during meiosis, we analyzed the immunoprecipitates of Rec11-TAP for phosphopeptides via mass spectrometry in two independent experiments. We found that Rec11 is phosphorylated on eight serine (S10, S22, S34, S43, S150, S439, S496 and S880) and two threonine (T60 and T70) residues during meiosis (Fig 2C).

To test the potential functional significance of Rec11 phosphorylation, we generated *rec11* mutants encoding alanine at these ten phosphorylation sites (allele *rec11-10A*) or the phosphomimetic aspartate at those positions (*rec11-10D*) (Fig 2C). The recombination-proficiency of the *rec11-10A* mutant was lower than that of wild type by factors of ~5–10, whereas that of the *rec11-10D* mutant was near that of wild type (Tables 3). These data indicate that phosphorylation of Rec11 at one or more of these sites is important for recombination. [Since in recombination assays the phosphomimetic aspartate is less deleterious than alanine (Table 3), we presume that the deficiency in the *rec11-10A* mutant results from reduced phosphorylation, although other structural deficiencies of Rec11 cannot be excluded. The abundance of both Rec11 mutant proteins during meiosis was similar to that of wild-type Rec11 (S4 Fig), indicating that the mutations do not significantly alter the stability of Rec11.] Because the recombination-deficiency of the *rec11-10A* mutants was not as great as that of the *rec11Δ* null mutant or of the *hhp1-as hhp2Δ* mutant in the absence of analog, Rec11 presumably has additional phosphorylation sites important for its promotion of recombination; these may be the residual sites phosphorylated in *hhp1-as hhp2Δ* (Fig 2). Alternatively, Rec11 may have phosphorylation-independent functions important for recombination, still present in the *rec11-10A* mutants but lacking in *rec11Δ*.

**Table 3:** Recombination defects in *hhp1-as hhp2∆* and *rec11* phosphorylation-site mutants resemble those in *rec8∆* and *rec11∆* mutants, and phosphomimetic *rec11* mutation partially suppresses *hhp1-as hhp2∆*.

*rec* and *hhp* genotype	Intragenic recombination^a^	Intergenic recombination^b^			
	*ade6-M26* x *ade6-52*	*ade6—arg1*	*lys4—his4*	*his4—arg4*	*ura1—lys7*
*+*	2200, 2100	65 (5)	10.7 (2)	64 (2)	605 cM^c^
*rec8∆*	7.9 ± 0.5 (5)	0.8 ^d^	1.3 (2)	11 (2)	28 (3)
*rec11∆*	6.9 ± 1.0 (5)	<1.2 (3)	2.7 ^d^	15 ^d^	76 ^d^
*hhp1-as hhp2∆*	28 ± 4.8 (5)	2.0 (8)	0.6 (2)	14 (2)	49 (4)
*hhp1-as hhp2∆ rec8∆*	3, <42	— ^e^	—	—	15 (3)
*hhp1-as hhp2∆ rec11∆*	—	—	—	—	37 (2)
*rec11-10A*	280, 285	16 (2)	—	—	—
*rec11-10D*	1300, 1400	64 (2)	—	—	—
*hhp1-as hhp2∆ rec11-10A*	7.0 ± 1.2 (6)	1.6 (6)	—	—	—
*hhp1-as hhp2∆ rec11-10D*	51 ± 9.6 (8)	5.3 (8)	—	—	—

^a^ Data, from (n) crosses, are the mean ± SEM of Ade^+^ recombinants per million viable spores. Individual data are given when two crosses were done.

^b^ Data are cM, calculated, using Haldane’s equation, from the observed recombinant fraction of data pooled from (n) crosses.

^c^ Nominal distance calculated from the genome average of 0.16 cM/kb between the markers [60].

^d^ From [18].

^e^ Not determined.

If phosphorylation of Rec11 depends on Hhp and is important for recombination, the *rec11* phosphomimetic mutation, *rec11-10D*, might suppress the loss of Hhp function in the *hhp1-as hhp2Δ* mutant. Indeed, *rec11-10D* partially suppressed *hhp1-as hhp2Δ* for both gene conversion (*ade6* intragenic recombination) and crossing-over (*ade6—arg1* intergenic recombination). The *rec11-10D* mutation slightly raised the Ade^+^ recombinant frequency in the *hhp1-as hhp2Δ* mutant from 28 to 51 (per million viable spores; p = 0.05 by one-tailed t-test) and the *ade6—arg1* crossover distance from 2.0 to 5.3 (cM; p < 0.001 by Fisher’s exact test) (Table 3). Suppression may be only partial owing to sites of phosphorylation other than those mutated in the *rec11-10D* mutant or to the phosphomimetic mutations being only partially effective, as is frequently observed [38,39].

As expected from the greater recombination-proficiency of *rec11-10D* than of *rec11-10A*, DSBs were more abundant in *rec11-10D* than in *rec11-10A* (Fig 1, middle two pairs of lanes in each panel). Only at one DSB hotspot, denoted “c” on *Not*I fragment C (Fig 1B, middle panel), were DSBs close to wild-type levels in *rec11-10A*; DSBs at this hotspot were also most abundant in *hhp1-as hhp2Δ*, *rec8Δ*, and *rec11Δ* (Fig 1) [14,18], showing that DSB formation at this hotspot is largely independent of these factors. At other hotspots, DSBs were slightly reduced in *rec11-10D* relative to wild type, but they were reduced more in *rec11-10A* though in most cases not to the level in *hhp1-as hhp2Δ*, *rec8Δ*, or *rec11Δ* (as noted above, DSB levels are indistinguishable in *rec8Δ* and *rec11Δ* [14,18]). These data are in accord with the recombination data in Table 3 —the *rec11-10A* mutation reduces both DSB formation and recombination more than the *rec11-10D* mutation but not as much as the *hhp1-as hhp2Δ*, *rec8Δ*, and *rec11Δ* mutations.

Because Hhp is required for cohesin removal and proper meiotic divisions [12,40], we tested whether Rec11 phosphorylation is required for these steps of meiosis. Although Rec8 phosphorylation is essential for its cleavage and removal and thus for segregation of chromosomes during meiosis I [12,40], meiotic divisions occurred normally in Rec11 phosphorylation-site mutants *10A* and *10D*, and no defect in Rec8 removal at the onset of anaphase I was observed (S5 Fig). Mutating one of the two separase cleavage sites on Rec8 (Rec8-RD1) leads to only a minor defect in chromosome segregation during meiosis [41]. We did not observe any further impairment of meiotic chromosome segregation when we analyzed cells expressing both Rec11-10A and Rec8-RD1 (S6 Fig). These results suggest that the Rec11 phosphorylation sites identified in our study are required for meiotic recombination but not for segregation of chromosomes during meiotic divisions.

### Hhp promotes chromosomal loading of LinE proteins Rec10 and Rec27

**Fig 3:**
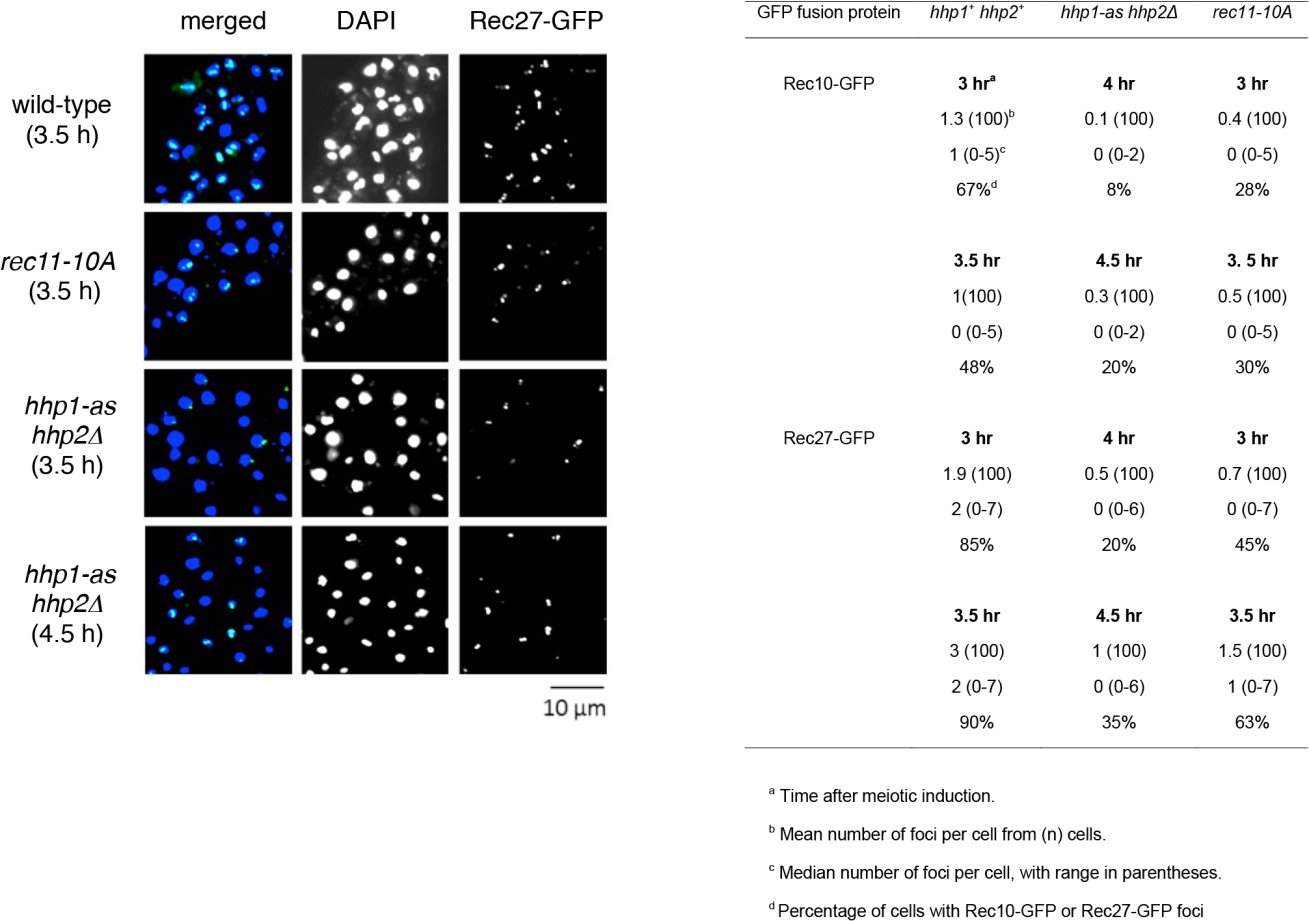
*hhp* and *rec11-10A* mutations reduce the number and intensity of foci of linear element proteins. Strains with the indicated mutations were induced for meiosis in the absence of an ATP analog. At the indicated times, cells were fixed, stained with DAPI, and examined by fluorescence microscopy for foci of the indicated GFP fusion protein (green) and DNA (blue). Representative cells with Rec27-GFP are shown, and quantification is given in the table below. Data for the *hhp1-as hhp2Δ* mutant were taken 1 hr later than for *hhp* ^*+*^ because replication is delayed by about 1 hr in the *hhp1-as hhp2Δ* mutant (S8 Fig). See also S6 Fig.

Rec8 and Rec11 appear to act before the LinE proteins, because the formation of LinEs or nuclear LinE protein foci largely depends on Rec8 and Rec11, but Rec8 focus-formation does not depend on LinE proteins [22]; Rec11 focus-formation has not, to our knowledge, been similarly tested. We therefore tested the dependence on Hhp of focus-formation by LinE proteins, using fluorescence microscopy of Rec10-GFP and Rec27-GFP, both of which are functional [14,19,20]. The number of foci formed by each protein and the fraction of cells with visible foci were significantly reduced in *hhp1-as hhp2Δ* in the absence of analog (p < 0.01 by Fisher’s exact test) as well as in *rec11-10A* (p < 0.01) (Fig 3 and S7 Fig). Comparison of either time point for wt (3 and 3.5 hr) with either time point for *hhp1-as hhp2∆* (4 and 4.5 hr) or for *rec11-10A* (3 and 3.5 hr) shows that the mutants have fewer foci and cells with foci. [We used later time points for the *hhp1-as hhp2∆* mutant because meiosis is delayed in the mutant (S8 Fig).] The residual levels were similar to those reported in *rec8Δ* and *rec11Δ* mutants, which behave similarly in other recombination-related assays [19,20]. In contrast, Rec11-GFP appeared to localize to the nucleus to nearly the same extent in wild type and in *hhp1-as hhp2Δ* (S9 Fig) but not in *rec8∆* (S10 Fig) as noted previously [42]. Rec11-GFP did not consistently form distinct foci, making quantification difficult. Nevertheless, the frequency of nuclei with Rec11-GFP fluorescence appeared to be similar in wild type and in *hhp1-as hhp2Δ*. These results show that the loading of LinE proteins depends on Hhp and likely on the phosphorylation of Rec11, which itself depends on Hhp (Fig 2).

## Discussion

Our results reveal that casein kinase 1 homologs in fission yeast, Hhp1 and Hhp2 (Hhp), have, in addition to their known substrate Rec8 [12,40], a second substrate that must be phosphorylated by Hhp during meiosis to promote DSB formation and recombination. We infer that the second substrate is Rec11, since inactivating Rec11 phosphorylation sites (by Ser or Thr → Ala changes) reduced recombination more in wild type cells than in Hhp-deficient cells (Table 3). Furthermore, phosphomimetic alterations (Ser or Thr → Asp) in Rec11 left the cells recombination-proficient, though not quite to the wild-type level, and partially suppressed the recombination-deficiency of Hhp-deficient cells (Table 3). Rec8 mutants lacking Hhp-dependent phosphorylation sites are deficient for cohesin cleavage [12,40] but are recombination-proficient (S3 Table). Conversely, Rec11 mutants lacking phosphorylation sites are deficient for recombination and DSB formation (Table 3; Fig 1 and S1 Fig) but are segregation-proficient (S5 Fig and S6 Fig). Therefore, our data combined with the cited published data show that Hhp phosphorylates Rec8 to regulate cohesin cleavage for proper chromosome segregation and mediates phosphorylation of Rec11 to activate DSB formation for recombination. A recent independent report drew the same conclusion [43]. These two actions of Hhp are separable, since lack of Rec8 phosphorylation leaves recombination (Rec11 action) intact (S3 Table) and reduction of Rec11 phosphorylation leaves chromosome segregation (Rec8 action) intact (S5 Fig and S6 Fig). Below, we discuss the implications of these findings for the mechanism of meiotic recombination and chromosome segregation, and their co-ordination. The conservation of Rec8 and Rec11 in most species suggests that these two separate roles of the cohesin subunits regulate meiotic chromosome dynamics in widely divergent species, including humans.

### Separable functions of Hhp during meiosis

We were surprised that the *hhp1-as* (M84G) ATP-analog-sensitive mutant had a dramatic, differential phenotype even in the absence of added analog (Tables 2 and 3; Figs 1, 2, and 3)—it strongly reduced recombination but had much less effect on viable spore yield or chromosome segregation (Tables 2 and 3). We infer that this mutation differentially alters the substrate specificity or activity of Hhp1 such that in the absence of analog the mutant Hhp1 adequately phosphorylates Rec8 but not Rec11 (at least not completely) (Tables 2 and 3; Figs 1, 2, and 3). This fortuitous result greatly aided our experiments because *hhp1Δ hhp2Δ* mutants grow poorly and have very low viable spore yields [26–28], whereas the *hhp1-as* mutants grow like wild type and have quite high viable spore yield in the absence of analog (Table 2). There are precedents for such differential inactivation of protein kinases by ATP-binding site mutations. For example, mutation of the “gatekeeper” residue in the ATP-binding site can reduce kinase activity even without analog present [29]. In addition, differential regulation of cellular events can arise from differential threshold levels for kinase activity [44,45].

During meiosis, Hhp plays major roles both in the timely removal of cohesin from chromosome arms at the onset of anaphase I, via phosphorylation of Rec8, and in DSB formation and recombination, via phosphorylation of Rec11. Since both of these processes are meiosis-specific, it is not surprising that we found physical interaction between Hhp and cohesin only in meiotic cells (S4 Table and S3 Fig); this association may aid the coordination of Rec8 cleavage and recombination. The abundance of *hhp1* and *hhp2* transcripts is greatly increased during meiosis [46], a feature consistent with Hhp playing especially important roles during meiosis. Hhp directly phosphorylates Rec8 [12,23], and it may directly phosphorylate Rec11 or activate another protein kinase that does; our data do not distinguish these possibilities, but it was recently reported that Hhp1 and Hhp2 can directly phosphorylate Rec11 [43]. Hhp clearly regulates separately two events essential for the formation of viable gametes and species propagation.

### A role for Hhp in the regional specificity of meiotic recombination

We found that the Hhp mutants (*hhp2Δ* coupled with *hhp1-as* or *hhp1Δ*) reduced recombination more in some intervals than in others (Tables 1, 2, and 3, and S3 Table), much like the region-dependent reductions by *rec8* and *rec11* mutations, including deletions [18,25]. Furthermore, *hhp1-as* abolished meiotic DSBs at some hotspots but not at others (Fig 1 and S1 Fig). The residual DSB patterns are reminiscent of those of cohesin and certain LinE mutants [14,18]. These observations lead us to propose that Rec11 phosphorylation is required for the loading of the putative Rec25-Rec27-Mug20 complex and Rec10 at DSB hotspots [14]. This proposal is consistent with the reduction, but not elimination, of Rec27-GFP foci in the *hhp1-as hhp2Δ* mutant (Fig 3 and S7 Fig). Rec11 phosphorylation seems not to be required for Rec11 to localize to the nucleus and possibly to load onto chromosomes, since Rec11-GFP nuclear localization was similar in wild type and in the *hhp1-as hhp2Δ* mutant (S9 Fig).

**Fig 4:**
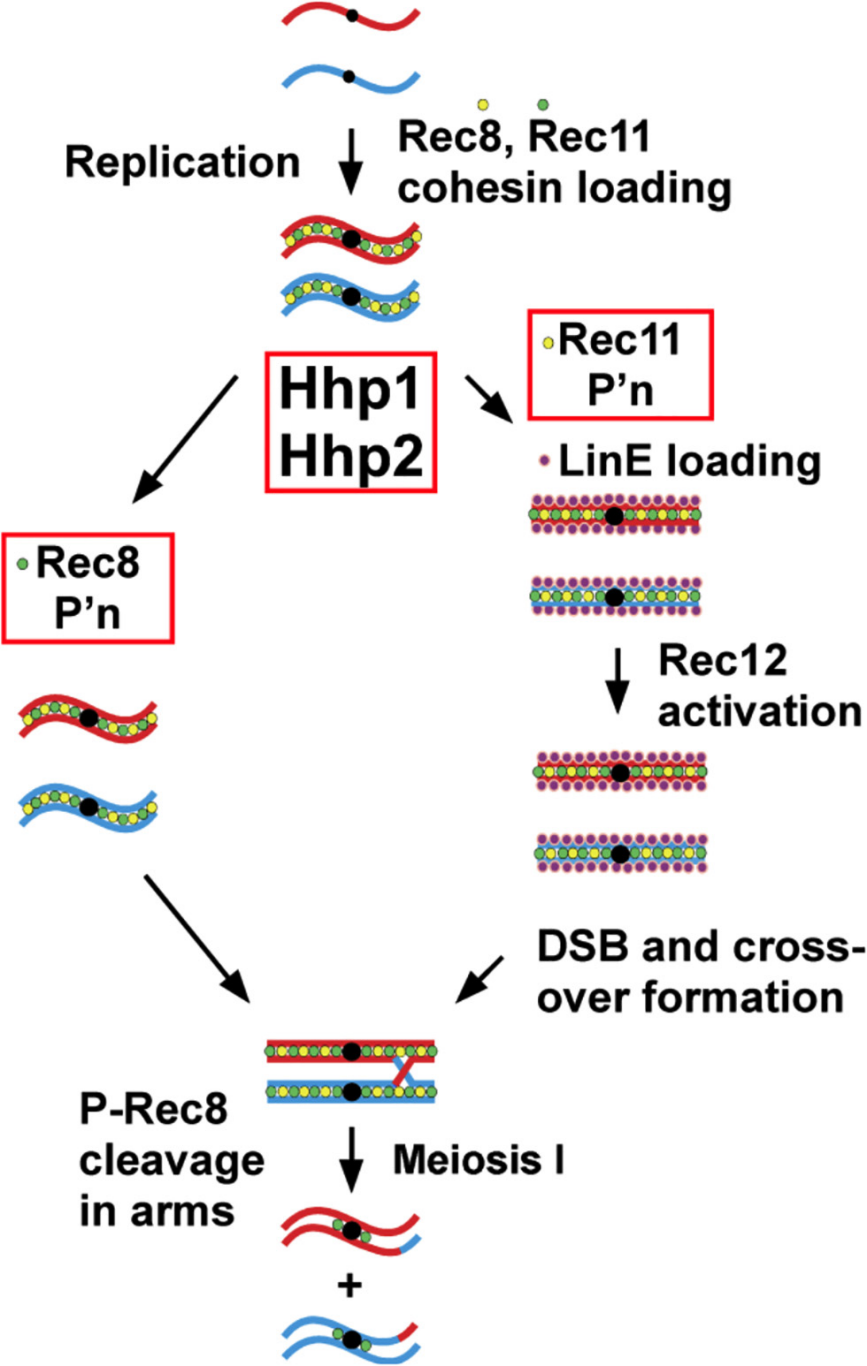
Scheme for dual action of casein kinase 1 (Hhp) on cohesin subunits to regulate meiotic chromosome segregation via Rec8 phosphorylation and to promote linear element formation, meiotic DSB formation, and recombination via Rec11 phosphorylation. Cohesin subunits Rec8 and Rec11 are loaded onto chromosomes prior to premeiotic replication, during which sister chromatid cohesion is established. Before, during, or after loading they are phosphorylated by casein kinase homologs Hhp1 and Hhp2. *Right*: Phosphorylation (P’n) of Rec11 leads to loading of the linear element (LinE) proteins Rec10, Rec25, Rec27, and Mug20, which in turn activate Rec12 (Spo11 homolog) and its partner proteins for DSB formation; DSBs lead to crossovers, which allow segregation of homologous centromeres, not sister centromeres, at the first meiotic division (MI). *Left*: Phosphorylation of Rec8 (generating P-Rec8) allows its cleavage, first in the arms, which allows segregation of homologous centromeres at MI; later, cleavage of Rec8 in the centromeres allows segregation of sister centromeres at MII. Rec8 is also needed for loading of Rec11 (S10 Fig) [42] and is thus indirectly required for DSB formation and recombination [14,18]. The precise timing of Rec8 and Rec11 phosphorylation is unknown. Rec8 and Rec11 (STAG3 functional homolog) and their phosphorylation may play similar roles in mammalian gametogenesis (see Discussion).

Our results, coupled with previous data [14], suggest the following scheme for formation of meiotic DSBs and crossovers (Fig 4). Rec8 is loaded onto chromosomes during S phase. Rec11 is concurrently or subsequently loaded in a Rec8-dependent manner (S10 Fig) [42] and then phosphorylated by Hhp, which allows the preferential loading of Rec25-Rec27-Mug20 at DSB hotspots. Rec10 is loaded at low levels independently of any of these proteins and, in their absence, activates the Rec12 complex to form DSBs at low level across the genome and at a few DSB hotspots. The Rec25-Rec27-Mug20 complex, whose loading at high levels at DSB hotspots depends on Rec8 and phosphorylated Rec11, along with Rec10 strongly activates the Rec12 complex to form DSBs at the hundreds of DSB hotspots dependent upon these proteins [14]. In this view, the main role of Hhp is to promote high level DSB formation at hotspots, which collectively account for about 70% of all DSBs and about half of all crossovers across the genome [47].

### Conservation of meiosis-specific cohesin subunits and their roles

The proteins discussed here are widely conserved across all eukaryotic phyla, including humans. To our knowledge, casein kinase homologs are present, often as multiple paralogs, in all eukaryotes examined, and all except apparently the protist *Tetrahymena thermophila* have meiosis-specific Rec8 cohesin subunits [7]. In only rare cases, such as the budding yeast *Saccharomyces cerevisiae* and *T*. *thermophila*, is there no identified meiosis-specific homolog of the Rec11 cohesin subunit [6,7,48,49]. Vertebrates harbor three Rec11-like STAG (stromal antigen) proteins, STAG1, STAG2, and STAG3. STAG3 is meiosis-specific, is required for meiotic sister chromatid cohesion and chromosome axis formation, and is closely related to the Rec11 protein studied here [50–52]. Murine STAG3 is phosphorylated during meiosis, and this modification appears to be required for meiotic progression [53]. STAG3-deficient mice, both male and female, are sterile and display severe meiosis I defects [54,55]. A *STAG3* frameshift mutation is apparently the cause of premature ovarian failure in humans [56]. Thus, our observations on Rec11 phosphorylation and its role in meiotic chromosome behavior are likely to pertain to meiosis and fertility in most species, including humans.

## Materials and Methods

### *S*. *pomb*e strains, mutant constructions, and genetic methods

Strains were constructed by standard meiotic crosses [57]; genotypes of strains and the figures and tables in which each was used are in S6 Table. Mutations were introduced into cloned genes using the QuikChangeII kit (Agilent Technologies), which were inserted into the genome by transformation to antibiotic-resistance [28]. Transformants were confirmed by PCR-based analysis and, in some cases, by nucleotide sequencing.

Meiotic crosses and analysis of random spore colonies were conducted as described [57]. The ATP analog 1-NM-PP1 (Toronto Research Chemicals) was added to the sporulation agar (SPA) at 50 μM; required amino acid, purine, and pyrimidine supplements were added at 100 μg/ml. Ade^+^ recombinant frequencies were determined by differential plating on yeast extract agar (YEA) with and without guanine (200 μg/ml). To determine intergenic recombinant frequencies, spore suspensions were plated on YEA; colonies were transferred with toothpicks to YEA supplemented with adenine (100 μg/ml), incubated overnight, and replicated to appropriate media to determine phenotypes. Recombinant frequencies were converted to genetic distance (cM) using Haldane’s equation [x = -½ *ln*(1-2R), where x is the distance in Morgans (M) and R is the recombinant frequency].

To prepare large meiotic cultures, cells were grown to log phase at 25° C in supplemented liquid minimal medium (EMM2), washed in H_2_O, resuspended in supplemented EMM2 without a nitrogen source, and incubated for 18–19 hr, at which time NH_4_Cl was added to 5 mg/ml and the temperature raised to 34° C to inactivate the Pat1-114 temperature-sensitive protein kinase [58]. Cells were harvested at appropriate times after induction and analyzed for DNA content by flow cytometry (to determine the time of replication) and for other features as described below.

### DNA and protein analysis

To determine DSBs, meiotic cells were washed, concentrated by centrifugation, and embedded in agarose plugs, which were sequentially treated with lytic enzymes, proteinase K, RNase, and appropriate restriction enzymes [58]. The digested DNA was separated by gel electrophoresis and analyzed by Southern blot hybridization.

To analyze phosphorylation and electrophoretic mobility of Rec11, cells expressing Rec11-TAP were arrested in G1 by nitrogen starvation, and meiosis was induced by shifting the culture to 34°C. Four hr later, cells from 20 mL of culture were concentrated by centrifugation, suspended in IPP150 buffer (50 mM Tris-HCl pH 8.0, 150 mM NaCl, 10% glycerol, 0.1% NP-40), and homogenized using glass beads (0.4–0.6 mm diameter). Extracted proteins were immunoprecipitated with IgG Sepharose 6 Fast Flow beads (GE Healthcare), treated with alkaline phosphatase (Thermo Scientific) as indicated and separated by electrophoresis through 5% polyacrylamide gels containing SDS (0.1%). Proteins were transferred to a PVDF membrane (Millipore), and Rec11-TAP and Hhp2-TAP were detected using rabbit antiperoxidase antibody linked to peroxidase (Dako; 1:30,000 dilution) in 0.1% PBS-T (8 gm NaCl, 0.2 gm KCl, 1.44 gm Na_2_HPO_4_, 0.24 gm KH_2_PO_4_, 1 mL Tween-20 per L). Hhp1-PK9 was detected using mouse-anti-PK (V5) antibody (Serotec; 1:2000 dilution) and goat anti-mouse IgG-HRP secondary antibody (Santa Cruz Biotechnology; 1:5000 dilution) in 0.1% PBS-T.

### Affinity purification of proteins

Cells from six-liter (mitotic) or fifteen-liter (meiotic) cultures of strains expressing TAP-tagged proteins were collected by centrifugation; meiotic cultures were harvested at 2.5–3.5 hr after induction of meiosis. Yeast cell powder was made from frozen pellets using a SamplePrep 6870 Freezer Mill (SPEX, Inc.). Proteins were extracted using IPP150 buffer containing complete protease and phosphatase inhibitors (Roche) and 1 mM PMSF (Sigma). All washing steps were performed in Poly-Prep columns (Bio-Rad) by gravity flow. IgG Sepharose™ 6 Fast Flow beads (500 μl; GE Healthcare) were washed with IPP150 buffer, mixed with protein extract, and rotated for 2 hr at 4°C. Beads were washed with IPP150 buffer and then with TEV cleavage buffer (TCB: 10 mM Tris-HCl pH 8.0, 150 mM NaCl, 10% glycerol, 0.1% NP-40, 0.5 mM EDTA, 1 mM DTT). Protein cleavage was performed in 2 ml of TCB buffer supplemented with 400 units of AcTEV protease (Life Technologies) for 2 hr at 16°C. The eluate (2 ml) was supplemented with 6 μl of 1 M CaCl_2_ and mixed with 6 ml of calmodulin binding buffer 1 (CBB1: 10 mM Tris-HCl pH 8.0, 150 mM NaCl, 10% glycerol, 0.1% NP-40, 1 mM imidazole, 1 mM Mg(OAc)_2_, 2 mM CaCl_2_, 10 mM β-mercaptoethanol). Calmodulin Sepharose 4B beads (150 μl; GE Healthcare) were washed with CBB1 buffer, added to a mixture of eluate and CBB1 buffer, and incubated for 2 hr at 4°C. The beads were washed with CBB1 and calmodulin binding buffer 2 (CBB2: 10 mM Tris-HCl pH 8.0, 150 mM NaCl, 1 mM Mg(OAc)_2_, 2 mM CaCl_2_, 1 mM β-mercaptoethanol). The proteins were step-eluted using one bed volume of elution buffer (EB: 10 mM Tris-HCl pH 8.0, 150 mM NaCl, 1 mM Mg(OAc)_2_, 2 mM EGTA, 1 mM β-mercaptoethanol). Eluted proteins were separated by SDS-PAGE and silver stained. Eluates from peak fractions were analyzed by LC-MS/MS as described in S1 Text and by Cipak et al. [59].

### Fluorescence microscopy

Haploid *pat1-114* cells were arrested by nitrogen starvation for 16 hr and released into meiosis at 34°C by inactivation of Pat1 and addition of nitrogen. Cells were harvested at the indicated time points after meiotic induction, fixed with 70% ethanol (Fig 3 and S7 Fig) or with 99.8% methanol (S9 Fig and S10 Fig), and stained with DAPI; nuclei were counted in 100 cells at each time point. The Rec10-GFP protein was visualized in unfixed cells (S7 Fig) as follows. Cells from 100 μl of culture were collected by centrifugation, washed once with water, and spread on a cover slip coated with poly-L-lysine. Slides with a drop of mounting medium containing DAPI were covered with the cover slips, and the cells were analyzed the next day. All images were from a single focal plane; out-of-focus cells were not scored. Images for Fig 3 and S7 Fig were obtained with a Zeiss Axio Imager.Z2 microscope equipped with a Plan Apochromat 63x/1.4 oil-immersion lens and an AxioCamMRm camera; images were analysed with ZEN 2011 software. Images for S9 Fig and S10 Fig were obtained with a Zeiss Apotome microscope equipped with a 100X /1.4 oil-immersion lens and were analyzed using Axiovision software. All inductions and localization analyses were performed at least twice. Haploid strains were analysed because diploid *hhp1-as hhp2∆* cells grow poorly even without analog and produce grossly abnormal asci.

## Supporting Information

S1 Text Supporting Information includes further information on strain constructions, protein analysis by mass spectrometry, and fluorescence microscopy. (DOCX)

S1 Fig Meiotic DNA breakage in *hhp*, *rec11*, and *rec8* mutants. Strains with the indicated mutations were induced for meiosis in the absence of an ATP analog. At the indicated times, DNA was extracted and analyzed by Southern blot hybridization. (Top) The 501 kb *Not*I fragment J on chromosome 1 was analyzed with a probe at its left end [60]. (Middle) The 1.5 Mb *Not*I fragment C on chromosome 2 was analyzed with a probe near its left end [18]. (Bottom) The 6.6 kb AflII fragment containing *ade6* on chromosome 3 was analyzed with a probe at its right end [61]. DSBs at the *ade6-3049* hotspot are spread over about 1 kb, indicated by the bar on the right and the markers (1.6, 2, 3, 4, 5, and 6 kb, bottom to top) between the panels for *rec11-10A* and *rec11-10D*.
(TIF)

S2 Fig Analysis of proteins bound to Hhp1, Hhp2, and Rec11 from mitotically growing cells and meiotically induced cells. Extracted proteins were collected on IgG beads, to which the indicated TAP-tagged proteins from the indicated strains bind. Bound proteins were separated by gel electrophoresis and stained with silver. MW, molecular mass standards.
(TIF)

S3 Fig Hhp1 and Hhp2 physically interact in meiosis. (A) (Top) Proteins extracted from cells induced for meiosis for the indicated times were analyzed for total Hhp1 and Hhp2 by gel electrophoresis and Western blotting using antibodies to the indicated proteins fused to Hhp1 or Hhp2. (Bottom) Proteins bound to IgG beads, which binds the TAP tag on Hhp2, were analyzed for Hhp1 by Western blotting with anti-PK9 antibody. (B) Cycling cells expressing Hhp1-PK9 alone (left panel) or both Hhp1-PK9 and Hhp2-TAP (right panel) were treated with bleomycin (2.0 μg/ml) for 2 hr. Proteins were extracted and analyzed as in (A).
(TIF)

S4 Fig Abundance and mobility of wild-type Rec11, Rec11-10A, and Rec11-10D mutant proteins. *pat1-114 rec11-TAP* cells (*hhp1* ^*+*^ *hhp2* ^*+*^ or *hhp1-as hhp2Δ*, as indicated) were induced for meiosis in medium containing (+) or lacking (–) 30 μM 1-NM-PP1, an ATP analog. Extracted proteins were treated with alkaline phosphatase (AP) (+) or not (–). Eighty μg of protein (determined by Bradford assay) were loaded per lane and separated by gel electrophoresis. Rec11-TAP protein was detected by western blotting using peroxidase anti-peroxidase antibody.
(TIF)

S5 Fig Meiotic divisions and Rec8 localization occur normally in *rec11-10A* and *rec11-10D* mutants. (A) *pat1–114* cells carrying *rec11* ^*+*^ (wt), *rec11-10A*, or *rec11-10D* were arrested by nitrogen starvation and released into meiosis at 34°C by inactivation of Pat1. Cells were harvested at the indicated times (hr) after meiotic induction and stained with DAPI; nuclei were counted in 100 cells per time point. The fraction of cells that contained one nucleus (1), two nuclei (2), or more than two nuclei (3) at the indicated times are shown. (B) The wild-type and the indicated mutant strains were sporulated on PMG-N plates for 18 hr, stained with DAPI and examined under the fluorescence microscope. The number of nuclei was scored in 100 asci. Whereas meiotic nuclear divisions were greatly inhibited in cells expressing a mutant version of Rec8 in which both separase cleavage sites were mutated (Rec8-RDRD), as reported previously [5], *rec11-10A* and *rec11-10D* mutants produced asci with four nuclei, similarly as the wild-type. (C) Wild-type, *rec11-10A* or *rec11-10D* mutant cells expressing Rec8-GFP were sporulated, fixed and stained with antibodies against tubulin and GFP. Nuclei were visualized by Hoechst staining. 100 late-anaphase cells with either a general nuclear Rec8-GFP signal or a weak nuclear Rec8-GFP focus (presumably Rec8-GFP at centromeric regions) were scored.
(TIF)

S6 Fig *rec11-10A* mutation does not impair meiotic chromosome segregation in *rec8-RD1* mutant cells. Segregation of chromosome I (*lys1-*GFP dots) was scored in the indicated strains. Cells were sporulated on PMG-N plates for 40 hr, stained with DAPI, and examined under a fluorescence microscope. Chromosome segregation was scored in at least 100 asci.
(TIF)

S7 Fig *hhp* mutations reduce the number and intensity of foci of linear element proteins. Strains with the indicated mutations were induced for meiosis in the absence of an ATP analog. At the indicated times, live cells with Rec10-GFP were stained with Hoechst 33342; cells with Rec27-GFP were fixed with methanol and stained with DAPI. Cells were examined by fluorescence microscopy for foci of the indicated GFP fusion protein and DNA. Cells that did not stain for DNA were not counted. Representative cells with Rec10-GFP or Rec27-GFP are shown. Quantification is given in the table, one experiment for Rec10-GFP and pooled data from two experiments with Rec27-GFP.
(TIF)

S8 Fig Meiosis is delayed in *hhp1-as hhp2∆* meiosis in the absence of analog. At the indicated times after meiotic induction, cells were assayed by flow cytometry for DNA content. Note that at 0 hr (at the time of meiotic induction) G1 cells (left peaks) outnumber G2 cells (right peaks). After replication the opposite is true.
(TIF)

S9 Fig Rec11-GFP is localized in the nuclei of *hhp* mutants nearly as frequently as in those of wild type. Cells with the indicated genotype were induced for meiosis in the absence of analog, fixed with methanol at the indicated time, stained with DAPI, and examined by differential interference contrast (DIC) microscopy for cells and by fluorescence microscopy for DNA (DAPI) and Rec11-GFP. Graphed data are the mean percent of cells at 4.5 hr with Rec11 foci from two experiments; error bars indicate the range. For wt 198 cells were scored; for *hhp* mutant 134 cells were scored.
(TIF)

S10 Fig Nuclear Rec11 foci are not visible in *rec8∆* mutant cells. Wild-type and *rec8∆* strains expressing Rec11-GFP were induced for meiosis. 3.5 hours after induction of meiosis, cells were fixed with methanol, stained with DAPI, and examined by differential interference contrast (DIC) microscopy for cells and by fluorescence microscopy for DNA (DAPI) and Rec11-GFP.
(TIF)

S1 Table Segregation of homologous centromeres during meiosis I in *hhp* mutants. (DOCX)

S2 Table Hhp is not required for meiotic recombination at an artificial DSB. (DOCX)

S3 Table Rec8 phosphorylation sites are not required for meiotic recombination or DSB formation. (DOCX)

S4 Table Hhp1 and Hhp2 physically interact with the cohesin complex during meiosis. (DOCX)

S5 Table Proteins co-purifying with Hhp1-TAP, Hhp2-TAP, and Rec11-TAP identified by mass spectrometry. (XLSX)

S6 Table *S*. *pombe* strains and genotypes. (DOCX)
