# Correction: Casein Kinase 1 and Phosphorylation of Cohesin Subunit Rec11 (SA3) Promote Meiotic Recombination through Linear Element Formation

**DOI:** 10.1371/journal.pgen.1005343

**Published:** 2015-06-24

**Authors:** The PLOS Genetics Staff

The last author’s affiliations are listed incorrectly on the website, but are correct in the downloadable PDF. The publisher apologizes for the error. Juraj Gregan is affiliated with:

**2.** Max F. Perutz Laboratories, University of Vienna, Vienna, Austria and,

**4.** Department of Genetics, Comenius University, Bratislava, Slovakia
