# Supplementary material for: Casein Kinase 1 and Phosphorylation of Cohesin Subunit Rec11 (SA3) Promote Meiotic Recombination through Linear Element Formation
Source: PLoS Genet. 2015 May 20;11(5):e1005225. doi: 10.1371/journal.pgen.1005225 (PMC4439085; doi:10.1371/journal.pgen.1005225)
Supplement: S1 Text — (DOCX) [file pgen.1005225.s001.docx]

**Supporting Information**

**Casein Kinase 1 and Phosphorylation of Cohesin Subunit Rec11 (SA3) Promote Meiotic Recombination through Linear Element Formation**

Naina Phadnis, Lubos Cipak, Silvia Polakova, Randy W. Hyppa, Ingrid Cipakova, Dorothea Anrather, Lucia Karvaiova, Karl Mechtler, Gerald R. Smith, and Juraj Gregan

**Additional Materials and Methods**

**Strain Construction**

Strains were constructed by standard transformations and meiotic crosses [[57](#_ENREF_57)]. New alleles of *rec11, hhp1,* and *hhp2* were constructed as follows.

Deletions of the *rec11, hhp1,* and *hhp2* genes were prepared according to Gregan et al. [[60](#_ENREF_60)], and tagging was performed according to Cipak et al. [[59](#_ENREF_59)].

To construct the *rec11-10A* and *rec11-10D* alleles, DNA flanking rec11 was PCR-amplified from genomic DNA using primers 5′- AAAATCTAGAcgccagtggacgaagagatgg-3′ and 5′-AAAACTCGAGcgatggtggttggttatggcaaag-3′ for the upstream region and primers 5′- AAAATCTAGAccttgcacaaaaaggtgttggttg-3′ and 5′- AAAAAGATCTccccgagggaaaagagataagaaagc-3′ for the downstream region. The products were digested with *Xba*I enzyme, ligated to each other, and cloned into a pClone-NatMX4 vector (EF101285) carrying drug-resistance markers for E. coli (ampicillin) and S. pombe (nourseothricin) using *Xho*I and *Bgl*II enzymes. The resulting pClone::NatMX4-rec11△ plasmid was recovered and amplified in E. coli, linearized by cutting with *Xba*I enzyme, and used to delete rec11 in a diploid strain JG11315. The deletion of the rec11 was confirmed by colony PCR.

To integrate *rec11* into the rec11*△* mutant strain, primers Rec11-fw 5’-ATATATGGATCCGTAGGCAGTAGCAGCAATGAAGC-3’ and Rec11-rev 5’- ATATATCCCGGGGAAAGCCACAGTACAAGTCGTGC-3’ were used to amplify *rec11* together with its flanking 5’- and 3’-regions and cloned into pClone::hphMX4 vector (EF101286) carrying drug-resistance markers for E. coli (ampicillin) and S. pombe (hygromycin B) using *BamH*I and *Sma*I enzymes. Site-directed mutagenesis (QuikChangeII Site Directed Mutagenesis Kit, Agilent Technologies, Inc.) was used to mutate the serine and threonine codons to phospho-null (Ala) and phospho-mimetic (Asp) codons using pClone-hphMX4-rec11 plasmid as a template. The prepared plasmids were linearized with *Bsu36*I enzyme and transformed into the *rec11∆* mutant using a LiOAc transformation protocol. The presence of the re-integrated *rec11* allele and *rec11(ST→A/D)* mutant alleles was confirmed by colony PCR.

**Mass Spectrometry Analysis of Proteins Immunoprecipitated with Hhp1-TAP and Hhp2-TAP**

Proteins that co-purified with Hhp1-TAP and Hhp2-TAP (see Materials and Methods) were prepared for mass spectrometry (MS) as previously described by [[59](#_ENREF_59)]. Eluted proteins were reduced with dithiothreitol (DTT), alkylated with iodoacetamide, and digested with trypsin at 37° C overnight. Digestion was stopped by addition of formic acid to 1%. Tryptic digests were separated on a nano reversed-phase HPLC column (Ultimate, Switchos, Famos; LC-Packings). Peptides were loaded onto a trapping column (PepMap C18, 300 µm × 5 mm) using 0.1% TFA at a flow rate of 20 µl/min. Bound peptides were eluted onto a 75 µm × 150 mm analytical column of the same material at a flow rate of 250 nl/min by applying a 1-hr linear gradient of acetonitrile in 0.1% formic acid. The LC column was coupled directly to the LTQ linear ion trap mass spectrometer (Thermo Fisher Scientific, Bremen, Germany) via a nano electrospray ion source (Proxeon, Odense, Denmark; now Thermo Fisher Scientific). The electrospray voltage was set to 1500 V. The mass spectrometer was operated in the data-dependent mode: 1 full scan (m/z 450-1600) was followed by MS/MS scans of the four most abundant ions. These ions were excluded from further selection for 20 s. The collision energy was set at 35%, the activation q value at 0.25, and the activation time at 30 ms.

Raw spectra were interpreted by Mascot 2.2.07 (Matrix Science Ltd, London, UK). The spectral data were searched against the Sanger *S. pombe* protein database with the following settings: tryptic specificity allowing two missed cleavages, carbamidomethylation of cysteine as static modification, and oxidation of methionine as variable modification. The peptide tolerance was set to 2 Da, and fragment tolerance to 0.8 Da. The result was filtered to 1% FDR using the Percolator algorithm integrated in Thermo Proteome Discoverer and the minimum peptide length was set to 7 amino acids.

**Mass Spectrometry Analysis of Proteins Immunoprecipitated with Rec11-TAP Using QExactive**

*NanoLC-MS Analysis*

The nano HPLC system used was an UltiMate 3000 HPLC RSLC nano system (Thermo Fisher Scientific) coupled to a QExactive mass spectrometer (Thermo Fisher Scientific), equipped with a Proxeon nanospray source (Proxeon). Peptides were loaded onto a PepMap trap column (C18, 5 mm × 300 μm ID, 5 μm particles, 100 Å pore size; Thermo Fisher Scientific) at a flow rate of 25 μL min^-1^ using 0.1% TFA as mobile phase. After 10 min, the trap column was switched in-line with a PepMap analytical column (C18, 500 mm × 75 μm ID, 3 μm particles, 100 Å pore size; Thermo Fisher Scientific). Peptides were eluted using a flow rate of 230 nL min^-1^ and a binary 3 hr gradient (total time including equilibration was 225 min). The gradient started with the mobile phases: 98% A (water/formic acid, 99.9/0.1, v/v) and 2% B (water/acetonitrile/formic acid, 19.92/80/0.08, v/v/v), increased to 35% B over the next 180 min, followed by a 5 min gradient to 90% B, remained there for 5 min and decreased in 2 min back to the gradient 98% A and 2% B for equilibration at 30°C for 20 min.

The QExactive mass spectrometer was operated in data-dependent mode, using a full scan (m/z range 350-1650, nominal resolution of 70,000, target value 1,000,000 followed by MS/MS scans of the 12 most abundant ions. MS/MS spectra were acquired using normalized collision energy 30%, isolation width of 2 and the target value was set to 50,000. Precursor ions selected for fragmentation (charge state 2 and higher) were put on a dynamic exclusion list for 10 s. Additionally, the underfill ratio was set to 20%, resulting in an intensity threshold of 20,000. The “peptide match” and “exclude isotopes” features were enabled.

*Data Analysis*

For peptide identification, the .RAW-files were loaded into Proteome Discoverer (version 1.4.0.288, Thermo Scientific), and the MS/MS spectra were searched using Mascot 2.2.07 (Matrix Science, London, UK) against the *S. pombe* protein sequence database. The following search parameters were used: Beta-methylthiolation of cysteine was set as a fixed modification, and oxidation of methionine and phosphorylation of serine, threonine and tyrosine were set as variable modifications. Monoisotopic masses were searched within unrestricted protein masses and no enzymatic specificity. The peptide mass tolerance was set to ± 5 ppm and the fragment mass tolerance to ± 0.03 Da. The maximal number of missed cleavages was set to 2. The result was filtered to 1% FDR using the Percolator algorithm integrated in Thermo Proteome Discoverer. The localization of the phosphorylation sites within the peptides was performed with the tool phosphoRS [[61](#_ENREF_61)]. For better visualization, the results of the searches were loaded into Scaffold (version 3.6.4, Proteome Software Inc.), using 1% false discovery rate as cutoff filter.

**Mass Spectrometry Analysis of Proteins Immunoprecipitated with Rec11-TAP using Velos**

*NanoLC-MS Analysis*

Analysis was as for the QExactive method above, except as follows. An LTQ OrbitrapVelos mass spectrometer (Thermo Fisher Scientific) was used in place of the QExactive mass spectrometer. Peptides were eluted using a flow rate of 230 nl min^-1^, and a binary 2 hr gradient (total time including equilibration was 165 min). The 180 min gradient was replaced with a 120 min gradient, which returned to 98% A and 2% B over 5 min.

The LTQ OrbitrapVelos was operated in data-dependent mode, using a full scan in the Orbitrap (m/z range 350-2000, nominal resolution of 60,000, target value 1,000,000) followed by MS/MS scans of the 12 most abundant ions in the linear ion trap. MS/MS spectra (normalized collision energy 35%; activation value q 0.25; activation time 10 ms; isolation width 2, target value 10,000) were acquired and subsequent activation was performed on fragment ions through multistage activation. The neutral loss mass list was therefore set to -98, -49, and -32.6 m/z. Precursor ions selected for fragmentation (charge state 2 and higher) were put on a dynamic exclusion list for 60 s. Additionally, singly-charged parent ions were excluded from selection for MS/MS experiments and the monoisotopic precursor selection feature was enabled.

*Data Analysis*

Data were analyzed as for the QExactive analysis, except that the fragment mass tolerance was set to ± 0.5 Da, the results were not filtered with the Percolator algorithm, and visualization used a minimum of two unique peptides per protein and a Mascot Score of at least 20 as cutoff filters.
