# Supplementary figures and images for: Casein Kinase 1 and Phosphorylation of Cohesin Subunit Rec11 (SA3) Promote Meiotic Recombination through Linear Element Formation
Source: PLoS Genet. 2015 May 20;11(5):e1005225. doi: 10.1371/journal.pgen.1005225 (PMC4439085; doi:10.1371/journal.pgen.1005225)

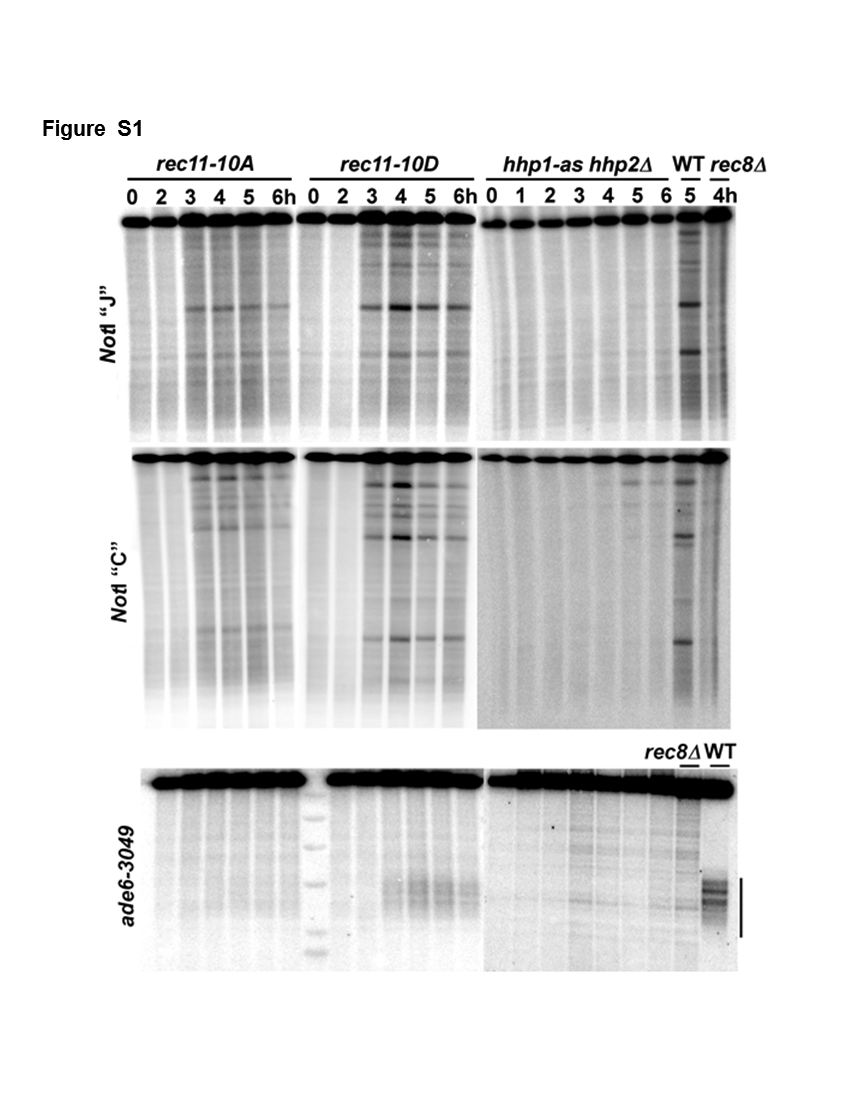

Supplement: S1 Fig — Strains with the indicated mutations were induced for meiosis in the absence of an ATP analog. At the indicated times, DNA was extracted and analyzed by Southern blot hybridization. (Top) The 501 kb NotI fragment J on chromosome 1 was analyzed with a probe at its left end [60]. (Middle) The 1.5 Mb NotI fragment C on chromosome 2 was analyzed with a probe near its left end [18]. (Bottom) The 6.6 kb AflII fragment containing ade6 on chromosome 3 was analyzed with a probe at its right end [61]. DSBs at the ade6-3049 hotspot are spread over about 1 kb, indicated by the bar on the right and the markers (1.6, 2, 3, 4, 5, and 6 kb, bottom to top) between the panels for rec11-10A and rec11-10D. (TIF) [file pgen.1005225.s002.tif]

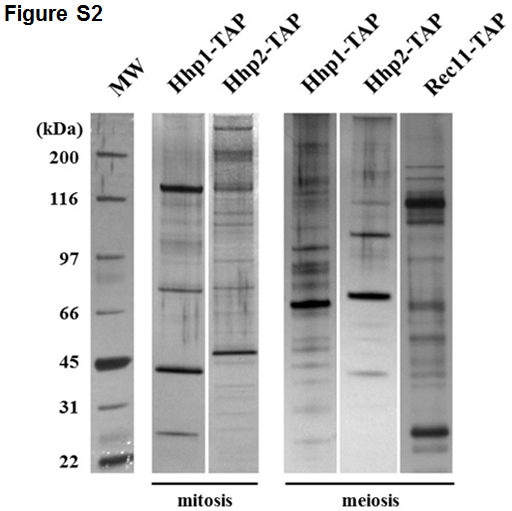

Supplement: S2 Fig — Extracted proteins were collected on IgG beads, to which the indicated TAP-tagged proteins from the indicated strains bind. Bound proteins were separated by gel electrophoresis and stained with silver. MW, molecular mass standards. (TIF) [file pgen.1005225.s003.tif]

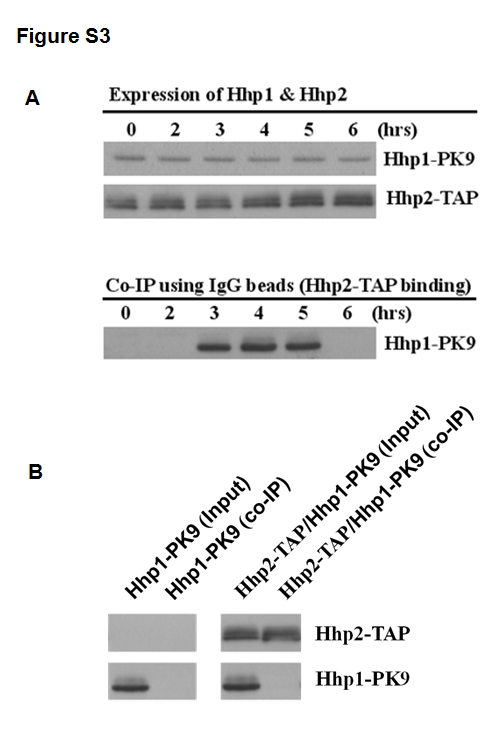

Supplement: S3 Fig — (A) (Top) Proteins extracted from cells induced for meiosis for the indicated times were analyzed for total Hhp1 and Hhp2 by gel electrophoresis and Western blotting using antibodies to the indicated proteins fused to Hhp1 or Hhp2. (Bottom) Proteins bound to IgG beads, which binds the TAP tag on Hhp2, were analyzed for Hhp1 by Western blotting with anti-PK9 antibody. (B) Cycling cells expressing Hhp1-PK9 alone (left panel) or both Hhp1-PK9 and Hhp2-TAP (right panel) were treated with bleomycin (2.0 μg/ml) for 2 hr. Proteins were extracted and analyzed as in (A). (TIF) [file pgen.1005225.s004.tif]

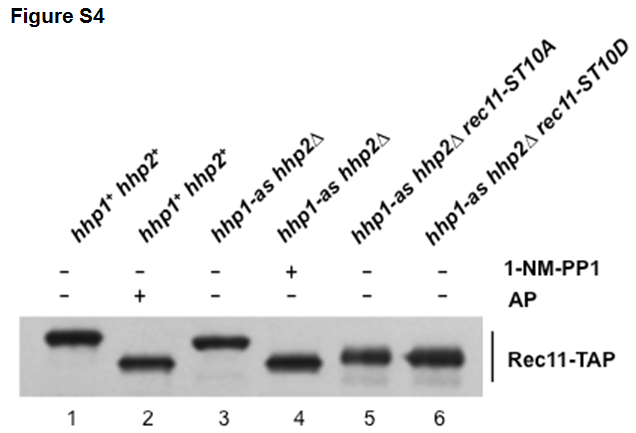

Supplement: S4 Fig — pat1-114 rec11-TAP cells (hhp1 + hhp2 + or hhp1-as hhp2Δ, as indicated) were induced for meiosis in medium containing (+) or lacking (–) 30 μM 1-NM-PP1, an ATP analog. Extracted proteins were treated with alkaline phosphatase (AP) (+) or not (–). Eighty μg of protein (determined by Bradford assay) were loaded per lane and separated by gel electrophoresis. Rec11-TAP protein was detected by western blotting using peroxidase anti-peroxidase antibody. (TIF) [file pgen.1005225.s005.tif]

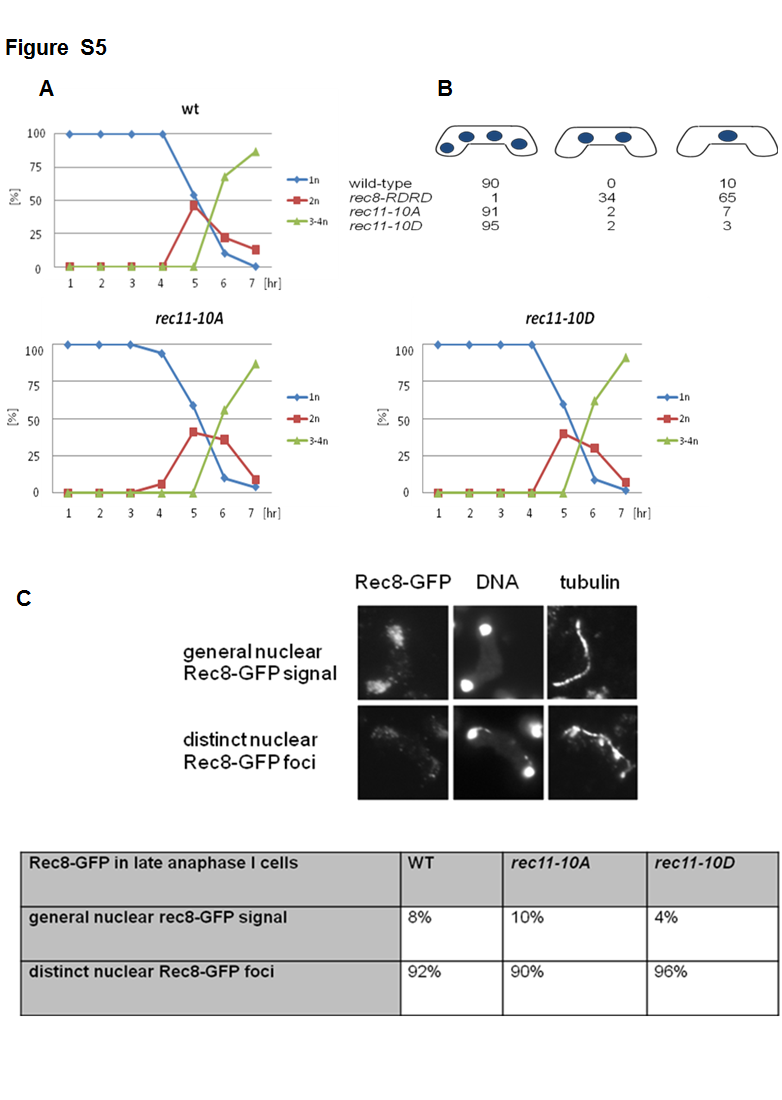

Supplement: S5 Fig — (A) pat1–114 cells carrying rec11 + (wt), rec11-10A, or rec11-10D were arrested by nitrogen starvation and released into meiosis at 34°C by inactivation of Pat1. Cells were harvested at the indicated times (hr) after meiotic induction and stained with DAPI; nuclei were counted in 100 cells per time point. The fraction of cells that contained one nucleus (1), two nuclei (2), or more than two nuclei (3) at the indicated times are shown. (B) The wild-type and the indicated mutant strains were sporulated on PMG-N plates for 18 hr, stained with DAPI and examined under the fluorescence microscope. The number of nuclei was scored in 100 asci. Whereas meiotic nuclear divisions were greatly inhibited in cells expressing a mutant version of Rec8 in which both separase cleavage sites were mutated (Rec8-RDRD), as reported previously [5], rec11-10A and rec11-10D mutants produced asci with four nuclei, similarly as the wild-type. (C) Wild-type, rec11-10A or rec11-10D mutant cells expressing Rec8-GFP were sporulated, fixed and stained with antibodies against tubulin and GFP. Nuclei were visualized by Hoechst staining. 100 late-anaphase cells with either a general nuclear Rec8-GFP signal or a weak nuclear Rec8-GFP focus (presumably Rec8-GFP at centromeric regions) were scored. (TIF) [file pgen.1005225.s006.tif]

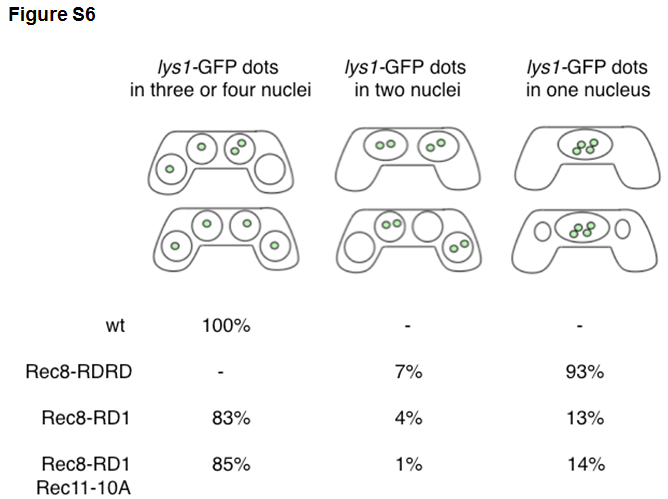

Supplement: S6 Fig — Segregation of chromosome I (lys1-GFP dots) was scored in the indicated strains. Cells were sporulated on PMG-N plates for 40 hr, stained with DAPI, and examined under a fluorescence microscope. Chromosome segregation was scored in at least 100 asci. (TIF) [file pgen.1005225.s007.tif]

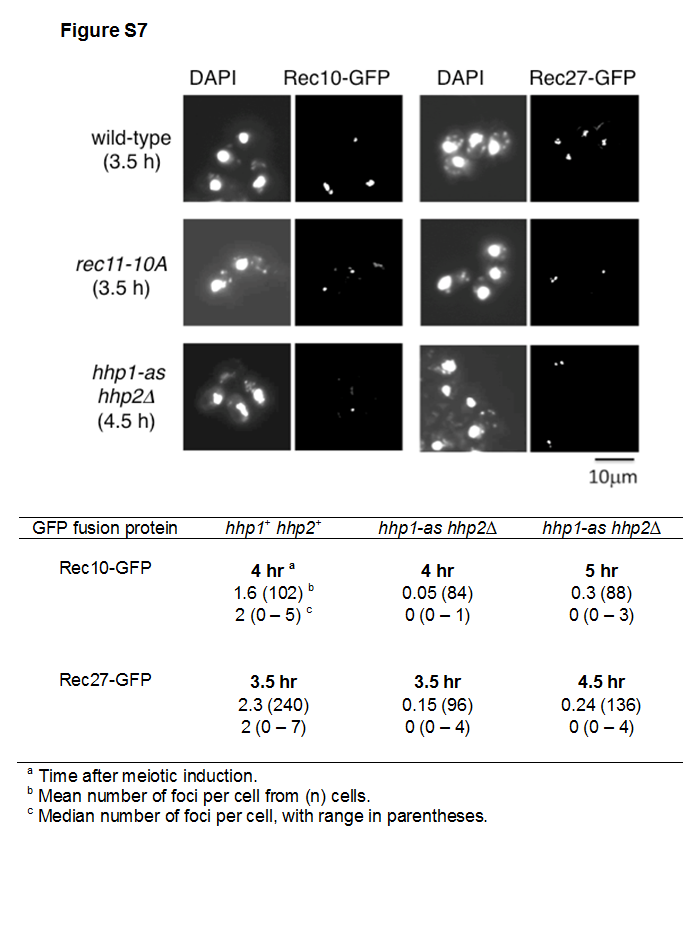

Supplement: S7 Fig — Strains with the indicated mutations were induced for meiosis in the absence of an ATP analog. At the indicated times, live cells with Rec10-GFP were stained with Hoechst 33342; cells with Rec27-GFP were fixed with methanol and stained with DAPI. Cells were examined by fluorescence microscopy for foci of the indicated GFP fusion protein and DNA. Cells that did not stain for DNA were not counted. Representative cells with Rec10-GFP or Rec27-GFP are shown. Quantification is given in the table, one experiment for Rec10-GFP and pooled data from two experiments with Rec27-GFP. (TIF) [file pgen.1005225.s008.tif]

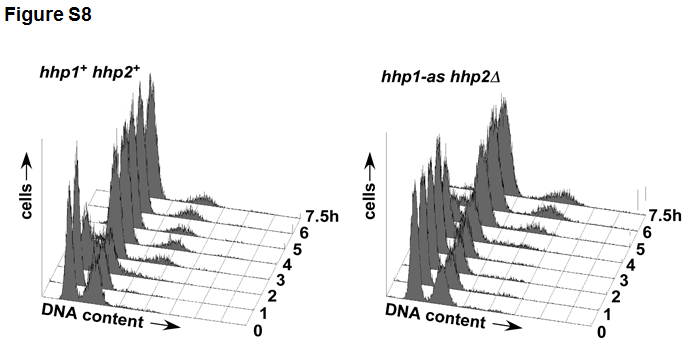

Supplement: S8 Fig — At the indicated times after meiotic induction, cells were assayed by flow cytometry for DNA content. Note that at 0 hr (at the time of meiotic induction) G1 cells (left peaks) outnumber G2 cells (right peaks). After replication the opposite is true. (TIF) [file pgen.1005225.s009.tif]

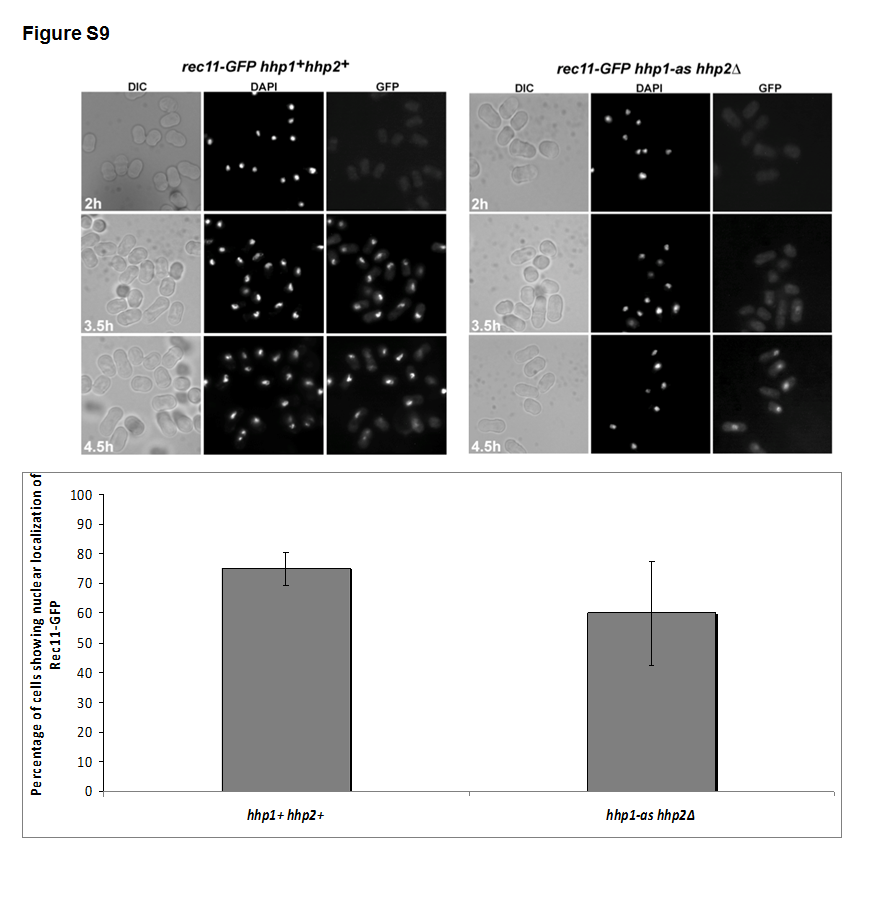

Supplement: S9 Fig — Cells with the indicated genotype were induced for meiosis in the absence of analog, fixed with methanol at the indicated time, stained with DAPI, and examined by differential interference contrast (DIC) microscopy for cells and by fluorescence microscopy for DNA (DAPI) and Rec11-GFP. Graphed data are the mean percent of cells at 4.5 hr with Rec11 foci from two experiments; error bars indicate the range. For wt 198 cells were scored; for hhp mutant 134 cells were scored. (TIF) [file pgen.1005225.s010.tif]

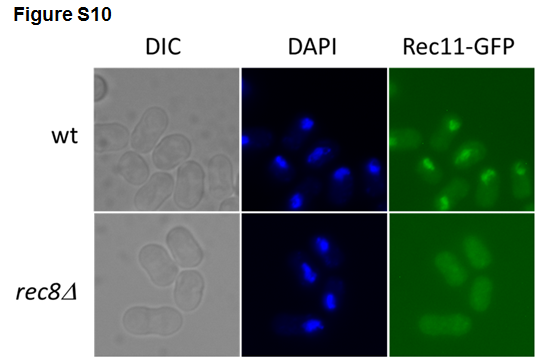

Supplement: S10 Fig — Wild-type and rec8∆ strains expressing Rec11-GFP were induced for meiosis. 3.5 hours after induction of meiosis, cells were fixed with methanol, stained with DAPI, and examined by differential interference contrast (DIC) microscopy for cells and by fluorescence microscopy for DNA (DAPI) and Rec11-GFP. (TIF) [file pgen.1005225.s011.tif]
