# Supplementary material for: Casein Kinase 1 and Phosphorylation of Cohesin Subunit Rec11 (SA3) Promote Meiotic Recombination through Linear Element Formation
Source: PLoS Genet. 2015 May 20;11(5):e1005225. doi: 10.1371/journal.pgen.1005225 (PMC4439085; doi:10.1371/journal.pgen.1005225)
Supplement: S1 Table — (DOCX) [file pgen.1005225.s012.docx]

**S1 Table. Segregation of homologous centromeres during meiosis I in *hhp* mutants**

| Genotype | ATP analog 1-NM-PP1 | Anaphase I homologs  properly segregated (%) | Anaphase I homolog  non-disjunction (%) |
| --- | --- | --- | --- |
| *hhp1 hhp2* |  |  |  |
| *+ +* | – | 100 | 0 |
| *+ +* | + | 100 | 0 |
| *as Δ* | – | 96 | 4 |
| *as Δ* | + | 83 | 17 |

Meiotic segregation of the centromere of chromosome 2 was scored in *h^90^ cen2*-GFP strains carrying the indicated *hhp* mutations and sporulated in the absence or presence of 1-NM-PP1 inhibitor. Cells were fixed and immunostained for tubulin (anaphase determination) and GFP (*cen2* positions). 100 anaphase I cells were examined by fluorescence microscopy.
