# Supplementary material for: Casein Kinase 1 and Phosphorylation of Cohesin Subunit Rec11 (SA3) Promote Meiotic Recombination through Linear Element Formation
Source: PLoS Genet. 2015 May 20;11(5):e1005225. doi: 10.1371/journal.pgen.1005225 (PMC4439085; doi:10.1371/journal.pgen.1005225)
Supplement: S2 Table — (DOCX) [file pgen.1005225.s013.docx]

**S2 Table. Hhp is not required for meiotic recombination at an artificial DSB**

| Relevant genotype | I-*Sce*I induced recombinants |
| --- | --- |
| *hhp1^+^ hhp2^+^* | 1350 ± 110 (13) |
| *hhp1-as hhp2∆* | 1470 ± 180 (11) |
| *rec8∆* | 500 ± 53 (10) |

Data, from (n) crosses, are the mean ± SEM of Ade^+^ recombinants/million viable spores in crosses between *ade6-3061* (I-*Sce*I cutting site) and *ade6-52* conducted in the absence of analog. In the absence of an I-*Sce*I cutting site, <10 Ade^+^ recombinants/million viable spores are observed [1].
