# Supplementary material for: Casein Kinase 1 and Phosphorylation of Cohesin Subunit Rec11 (SA3) Promote Meiotic Recombination through Linear Element Formation
Source: PLoS Genet. 2015 May 20;11(5):e1005225. doi: 10.1371/journal.pgen.1005225 (PMC4439085; doi:10.1371/journal.pgen.1005225)
Supplement: S3 Table — (DOCX) [file pgen.1005225.s014.docx]

**S3 Table. Rec8 phosphorylation sites are not required for meiotic recombination or DSB formation**

| Relevant genotype | Ade^+^ recombinants per million viable spores (*ade6-M26* x *ade6-52*) | *ade6 – arg1* recombinants (%) | Meiotic DSBs on *Not*I fragments C, D, and J |
| --- | --- | --- | --- |
| *rec8^+^* | 2500 ± 360 | 38 | Yes |
| *rec8-S412A* ^a^ | 3700 | 42 | Yes |
| *rec8-N12A* ^a^ | 2200 | 41 | Not assayed |
| *rec8-N12A S412A* | 2700 | 29 | Not assayed |
| *rec8-7A* ^a^ | 6600 ± 1300 | 28 | Yes |
| *rec8-17A* ^b^ | 2400 ± 260 | 35 | Not assayed |
| *rec8-17A S412A* | 2400 ± 130 | 33 | Not assayed |

Data (mean ± SEM) are from three to six crosses, except for *rec8-S412A, rec8-N12A,* and *rec8-N12A S412A* (one cross each).

^a^ Described in [2].

^b^ Described in [3].
