# Supplementary material for: Casein Kinase 1 and Phosphorylation of Cohesin Subunit Rec11 (SA3) Promote Meiotic Recombination through Linear Element Formation
Source: PLoS Genet. 2015 May 20;11(5):e1005225. doi: 10.1371/journal.pgen.1005225 (PMC4439085; doi:10.1371/journal.pgen.1005225)
Supplement: S4 Table — (DOCX) [file pgen.1005225.s015.docx]

**S4 Table. Hhp1 and Hhp2 physically interact with the cohesin complex during meiosis**

| **Cycling cells**  **PSMs/shared spectra** | | **Meiotic cells**  **PSMs/shared spectra** | | |
| --- | --- | --- | --- | --- |
| **Hhp1-TAP** | **Hhp2-TAP** | **Hhp1-TAP** | **Hhp2-TAP** | **Rec11-TAP** |
| Hhp1 (166/5) | Hhp1 (1/1) | Hhp1 (281/48) | Hhp1 (195/38) | Hhp1 (1/1) |
| Hhp2 (23/5) | Hhp2 (80/1) | Hhp2 (139/48) | Hhp2 (184/38) | Hhp2 (1/1) |
| - | - | Psm1 (8) | Psm1 (25) | Psm1 (94) |
| - | - | Psm3 (5) | Psm3 (19) | Psm3 (93) |
| - | - | Rec8 (2) | Rec8 (6) | Rec8 (54) |
| - | - | - | - | Rec11 (1824) |

Diploid *pat1-114* cells carrying the indicated TAP-tagged protein were induced for meiosis or grown to log phase (cycling cells). TAP-tagged proteins were immunoprecipitated (S2 Figure) and analyzed by mass spectrometry. Additional proteins not listed here were also detected (S5 Table). Values indicate the number of identified peptide-spectrum matches (PSMs). In addition, spectra shared between Hhp1 and Hhp2 are indicated for Hhp1 and Hhp2 proteins.
