# Supplementary material for: Casein Kinase 1 and Phosphorylation of Cohesin Subunit Rec11 (SA3) Promote Meiotic Recombination through Linear Element Formation
Source: PLoS Genet. 2015 May 20;11(5):e1005225. doi: 10.1371/journal.pgen.1005225 (PMC4439085; doi:10.1371/journal.pgen.1005225)
Supplement: S6 Table — (DOCX) [file pgen.1005225.s017.docx]

**S6 Table. *S. pombe* strains and genotypes**

| Strain number | Genotype | Used in |
| --- | --- | --- |
| GP13 | *h^-^ ade6-52* | Table 3 |
| GP14 | *h^+^ ade6-52* | Tables 3 and S3 |
| GP23 | *h^-^ ade6-M26* | Table 3 |
| GP127 | *h^-^ ade6-M26 arg1-14* | Table 3 |
| GP720 | *h^-^ ade6-52 ura4-294 leu1-32* | Table 2 |
| GP1293 | *h^+^ ade6-M26 arg1-14* | Table 3 |
| GP1456 | *h^-^ ade6-52 leu1-32 ura4-294 rec12-152::LEU2* | Table 2 |
| GP1459 | *h^+^ ade6-M26 leu1-32 ura4-294 rec12-152::LEU2* | Table 2 |
| GP2183 | *h^+^ ade6-M26 ura4-294 leu1-32* | Table 2 |
| GP2823 | *h^-^ ade6-52 leu1-32 ura4-D18 rec8-176::kanMX6* | Table 3 |
| GP4078 | *h^-^ smt0 ade6-52 leu1-32 ura4-D18* | Table S3 |
| GP4256 | *h^-^ade6-3049 pat1-114 rad50S* | Figs. 1 and S1 |
| GP4432 | *h^+^ ade6-3049 rec8-176::kanMX6 rad50S pat1-114 end1-458* | Figs. 1 and S1 |
| GP4594 | *h^-^ ade6-52 rec11::kanMX6* | Table 3 |
| GP4595 | *h^+^ ade6-M26 arg1-14 rec11::kanMX6* | Table 3 |
| GP4913 | *h^+^ ade6-M26 ura4-D18 arg1-14 rec8-176::kanMX6* | Tables 3 and S2 |
| GP5201 | *h^-^ smt0 ade6-3061 leu1-32 ura4-D18 rec12-172::I-SceI* | Table S2 |
| GP6170 | *h^+^ ade6-52 ura1-61 arg3-124 lys7-1* | Table 3 |
| GP6173 | *h^-^ ade6-52 ura1-61 arg3-124 lys7-1 rec8-176::kanMX6* | Table 3 |
| GP6178 | *h^+^ ade6-M26 rec8-176::kanMX6* | Table 3 |
| GP6265 | *h^-^ ade6-216 pat1-114 rec11-GFP::kanMX6* | Figs. S9 and S10 |
| GP6582 | *h^+^ ade6-52 leu1-32 ura4-D18 rec12-172::I-SceI* | Table S2 |
| GP6718 | *h^-^/h^-^ ade6-3049/ade6-3049 pat1-114/pat1-114 rec8-176::kanMX6/rec8-176::kanMX6 rec12-201::6His-2FLAG/rec12-201::6His-2FLAG lys4-95/his4-239* | Figs. 1 and S1 |
| GP6803 | *h^-^ ade6-3049 pat1-114 rec27-205-GFP::kanMX6* | Fig. S7 |
| GP6957 | *h^-^ rec10-203-GFP::kanMX6 pat1-114* | Figs. S7 and S8 |
| GP7421 | *h^+^ hhp1::natMX4 hhp1-as(M84G)::hphMX4 hhp2::kanMX6* | Table 2 |
| GP7422 | *h^-^ ade6-3049 pat1-114 rad50S hhp1::natMX4 hhp1-as(M84G)::hphMX4 hhp2::kanMX6* | Figs. 1 and S1 |
| GP7423 | *h^-^ ade6-3049 pat1-114 hhp1::natMX4 hhp1-as(M84G)::hphMX4 hhp2::kanMX6* | Figs. 1 and S1 |
| GP7433 | *h^+^ ade6-3061 leu1-32 rec12-172::I-SceI hhp1::natMX4 hhp1-as(M84G)::hphMX4 hhp2::kanMX6* | Table S2 |
| GP7434 | *h^-^ ade6-52 leu1-32 ura4-D18 rec12-172::I-SceI hhp1::natMX4 hhp1-as(M84G)::hphMX4 hhp2::kanMX6* | Table S2 |
| GP7441 | *h^-^ ade6-3049 pat1-114 rec8-FLAG-Tspo5::natMX4* | Table S3 |
| GP7443 | *h^-^ ade6-3049 pat1-114 rec8-S412A-FLAG-Tspo5::natMX4* | Table S3 |
| GP7445 | *h^+^ ade6-3049 pat1-114 rad50S rec8-7A-FLAG-Tspo5::natMX4* | Table S3 |
| GP7543 | *h^+^ ade6-52 hhp1::natMX4 hhp1-as(M84G)::hphMX4 hhp2::kanMX6* | Table 3 |
| GP7544 | *h^-^ ade6-52 hhp1::natMX4 hhp1-as(M84G)::hphMX4 hhp2::kanMX6* | Table 2 |
| GP7545 | *h^-^ade6-M26 hhp1::natMX4 hhp1-as(M84G)::hphMX4 hhp2::kanMX6 arg1-14* | Table 3 |
| GP7546 | *h^-^ leu1-32 hhp1::natMX4 hhp1-as(M84G)::hphMX4 hhp2::kanMX6 rec12-152::LEU2* | Table 2 |
| GP7547 | *h^-^ hhp1::natMX4 hhp1-as(M84G)::hphMX4 hhp2::kanMX6 rec12-152::LEU2* | Table 2 |
| GP7548 | *h^+^ leu1-32 hhp1::natMX4 hhp1-as(M84G)::hphMX4 hhp2::kanMX6 rec12-152::LEU2* | Table 2 |
| GP7587 | *h^+^ ade6-M26 arg1-14 rec8-FLAG-Tspo5::natMX4* | Table S3 |
| GP7590 | *h^-^ ade6-52 rec8-FLAG-Tspo5::natMX4* | Table S3 |
| GP7591 | *h^+^ ade6-M26 arg1-14 rec8-S412A-FLAG-Tspo5::natMX4* | Table S3 |
| GP7593 | *h^-^ ade6-52 rec8-S412A- FLAG-Tspo5::natMX4* | Table S3 |
| GP7605 | *h^-^ ade6-52 ura4-D18 rec8::kanMX6<<ura4^+^<<Prec8-rec8N12A-GFP::kanMX6* | Table S3 |
| GP7606 | *h^+^ ade6-M26 arg1-14 ura4-D18 rec8::kanMX6<<ura4^+^<<Prec8-rec8N12A::GFP-kanMX6* | Table S3 |
| GP7607 | *h^90^ ade6-52 ura4-D18 rec8::kanMX6<<ura4^+^<<Prec8-rec8-N12A-S412A-GFP::kanMX6* | Table S3 |
| GP7608 | *h^+^ ade6-M26 arg1-14 ura4-D18 rec8::kanMX6<<ura4^+^<<Prec8-rec8-N12A-S412A-GFP::kanMX6* | Table S3 |
| GP7633 | *h^-^ ade6-M26 arg1-14 rec8-7A-FLAG-Tspo5::natMX4* | Table S3 |
| GP7634 | *h^+^ ade6-52 rec8-7A-FLAG-Tspo5::natMX4* | Table S3 |
| GP7638 | *h^-^ ade6-3049 pat1-114 rec27-205-GFP::kanMX6 hhp1::natMX4 hhp1-as(M84G)::hphMX4 hhp2::kanMX6* | Fig. S7 |
| GP7672 | *h^+^ ade6-52 arg4-55 his4-239 lys4-95* | Table 3 |
| GP7674 | *h^-^ ade6-52 arg4-55 his4-239 lys4-95 rec8-176::kanMX6* | Table 3 |
| GP7676 | *h^+^ ade6-M26 hhp1::natMX4 hhp1-as(M84G)::hphMX4 hhp2::kanMX6* | Table 3 |
| GP7677 | *h^-^ ade6-M26 hhp1::natMX4 hhp1-as(M84G)::hphMX4 hhp2::kanMX6* | Table 3 |
| GP7679 | *h^-^ade6-52 arg4-55 lys4-95 his4-239 hhp1::natMX4 hhp1-as(M84G)::hphMX4 hhp2::kanMX6* | Table 3 |
| GP7680 | *h^+^ ade6-52 arg4-55 lys4-95 his4-239 hhp1::natMX4 hhp1-as(M84G)::hphMX4 hhp2::kanMX6* | Table 3 |
| GP7681 | *h^+^ ade6-52 ura1-61 lys7-1 rec8-176::kanMX6 hhp1::natMX4 hhp1-as(M84G)::hphMX4 hhp2::kanMX6* | Table 3 |
| GP7682 | *h^+^ ade6-52 ura1-61 lys7-1 arg3-124 hhp1::natMX4 hhp1-as(M84G)::hphMX4 hhp2::kanMX6* | Table 3 |
| GP7683 | *h^-^ ade6-M26 hhp1::natMX4 hhp1-as(M84G)::hphMX4 hhp2::kanMX6 rec8-176::kanMX6* | Table 3 |
| GP7691 | *h^-^ ade6-3061 leu1-32 ura4-D18 rec8-176::kanMX6 rec12-172::I-SceI* | Table S2 |
| GP7692 | *h^+^ ade6-52 leu1-32 ura4-D18 rec8-176::kanMX6 rec12-172::I-SceI* | Table S2 |
| GP7747 | *h^-^ ade6-M26 arg1-14* | Table S3 |
| GP7754 | *h^+^ ade6-M26 ura4-D18 arg1-14 rec8-176::kanMX6 rec8-17AS412A-ura4^+^::kanMX6* | Table S3 |
| GP7755 | *h^-^ ade6-52 ura4-D18 leu1-32 rec8-176::kanMX6 rec8-17AS412A-ura4^+^::kanMX6* | Table S3 |
| GP7781 | *h? ade6-216 pat1-114 hhp1::natMX4 hhp1-as(M84G)::hphMX4 hhp2::kanMX6 rec11-GFP::kanMX6* | Fig. S9 |
| GP7821 | *h^-^ rec11::kanMX6 hhp1::natMX4 hhp1-as(M84G)::hphMX4 hhp2::kanMX6* | Table 3 |
| GP7822 | *h^+^ ade6-52 rec11::kanMX6 ura1-61 lys7-1 arg3-124 hhp1::natMX4 hhp1-as(M84G)::hphMX4 hhp2::kanMX6* | Table 3 |
| GP7878 | *h^-^ ade6-52 rec11::kanMX6 rec11^+^::hphMX4* | Table 3 |
| GP7879 | *h^+^ ade6-M26 arg1-14 rec11::kanMX6 rec11^+^::hphMX4* | Table 3 |
| GP7915 | *h^-^ ade6-3049 pat1-114 rec10-203-GFP::kanMX6 hhp1::natMX4 hhp1-as(M84G)::hphMX4 hhp2::kanMX6* | Figs. S7 and S8 |
| GP7953 | *h^-^ ade6-216 pat1-114 rec11-GFP::kanMX6 rec8-176::kanMX6* | Figs. S6 and S10 |
| GP7979 | *h^-^ ade6-M26 arg1-14 rec8::natMX4 rec8-ST17A-ura4^+^::natMX4* | Table S3 |
| GP7980 | *h^+^ ade6-52 rec8::natMX4 rec8-ST17A-ura4^+^::natMX4* | Table S3 |
| GP8157 | *h^-^ ade6-52 rec11::kanMX6 rec11-10A::hphMX4* | Table 3 |
| GP8158 | *h^-^ade6-52 rec11::kanMX6 rec11-10D::hphMX4* | Table 3 |
| GP8159 | *h^+^ ade6-M26 arg1-14 rec11::kanMX6 rec11-10A::hphMX4* | Table 3 |
| GP8160 | *h^+^ ade6-M26 arg1-14 rec11::kanMX6 rec11-10D::hphMX4* | Table 3 |
| GP8246 | *h^-^ ade6-M26 arg1-14 rec11::kanMX6 rec11-10A::hphMX4 hhp1::natMX4 hhp1-as(M84G)::hphMX4 hhp2:kanMX6* | Table 3 |
| GP8247 | *h^+^ ade6-M26 arg1-14 rec11::kanMX6 rec11-10A::hphMX4 hhp1::natMX4 hhp1-as(M84G)::hphMX4 hhp2:kanMX6* | Table 3 |
| GP8248 | *h^-^ ade6-52 rec11::kanMX6 rec11-10A::hphMX4 hhp1::natMX4 hhp1-as(M84G)::hphMX4 hhp2::kanMX6* | Table 3 |
| GP8249 | *h^+^ ade6-52 rec11::kanMX6 rec11-10A::hphMX4 hhp1::natMX4 hhp1-as(M84G)::hphMX4 hhp2::kanMX6* | Table 3 |
| GP8250 | *h^+^ ade6-52 rec11::kanMX6 rec11-10D::hphMX4 hhp1::natMX4 hhp1-as(M84G)::hphMX4 hhp2::kanMX6* | Table 3 |
| GP8251 | *h^-^ ade6-M26 arg1-14 rec11::kanMX6 rec11-10D::hphMX4 hhp1::natMX4 hhp1-as(M84G)::hphMX4 hhp2::kanMX6* | Table 3 |
| GP8451 | *h^+^ ade6-3049 pat1-114 rad50S rec11::kanMX6 rec11-10A::hphMX4* | Figs. 1 and S1 |
| GP8452 | *h^+^ ade6-3049 pat1-114 rad50S rec11::kanMX6 rec11-10D::hphMX4* | Figs. 1 and S1 |
| JK100 | *h^+^ arg3A ade6-M26 arg3-D4 ura4-D18* | Table 1 |
| JK101 | *h^-^ ade6-469 tps16-23 arg3-D4* | Table 1 |
| JK102 | *h^+^ arg3A ade6-M26 arg3-D4 ura4-D18 hhp1::natMX4 hhp2::kanMX6* | Table 1 |
| JK103 | *h^-^ ade6-469 tps16-23 arg3-D4 hhp1::natMX4 hhp2::kanMX6* | Table 1 |

| JK104 | *h^+^* | Table 1 |
| --- | --- | --- |
| JK105 | *h^-^ ade1-40* | Table 1 |
| JK106 | *h^+^ hhp1::natMX4* | Table 1 |
| JK107 | *h^-^ ade1-40 hhp1::natMX4* | Table 1 |
| JK108 | *h^+^ hhp2::kanMX6* | Table 1 |
| JK109 | *h^-^ ade1-40 hhp2::kanMX6* | Table 1 |
| JK110 | *h^+^ hhp1::natMX4 hhp2::kanMX6* | Table 1 |
| JK111 | *h^-^ ade1-40 hhp1::natMX4 hhp2::kanMX6* | Table 1 |
| JK112 | *h^+^ lys4-95* | Table 1 |
| JK113 | *h^-^ ade1-40* | Table 1 |
| JK113 | *h^+^ lys4-95 hhp1::natMX4* | Table 1 |
| JK114 | *h^-^ ade1-40 hhp1::natMX4* | Table 1 |
| JK115 | *h^+^ lys4-95 hhp2::kanMX6* | Table 1 |
| JK116 | *h^-^ ade1-40 hhp2::kanMX6* | Table 1 |
| JK117 | *h^+^ lys4-95 hhp1::natMX4 hhp2::kanMX6* | Table 1 |
| JK118 | *h^-^ ade1-40 hhp1::natMX4 hhp2::kanMX6* | Table 1 |
| JK119 | *h^-^ ade6-M26* | Table 1 |
| JK120 | *h^+^ ade6-469* | Table 1 |
| JK121 | *h^-^ ade6-M26 hhp1::natMX4* | Table 1 |
| JK122 | *h^+^ ade6-469 hhp1::natMX4* | Table 1 |
| JK123 | *h^-^ ade6-M26 hhp2::kanMX6* | Table 1 |
| JK124 | *h^+^ ade6-469 hhp2::kanMX6* | Table 1 |
| JK125 | *h^-^ ade6-M26 hhp1::natMX4 hhp2::kanMX6* | Table 1 |
| JK126 | *h^+^ ade6-469 hhp1::natMX4 hhp2::kanMX6* | Table 1 |
| JK127 | *h^-^ ade6-M375* | Table 1 |
| JK128 | *h^+^* *ade6-469* | Table 1 |
| JK129 | *h^-^ ade6-M375 hhp1::natMX4* | Table 1 |
| JK130 | *h^+^* *ade6-469 hhp1::natMX4* | Table 1 |
| JK131 | *h^-^ ade6-M375 hhp2::kanMX6* | Table 1 |
| JK132 | *h^+^* *ade6-469 hhp2::kanMX6* | Table 1 |
| JK133 | *h^-^ ade6-M375 hhp1::natMX4 hhp2::kanMX6* | Table 1 |
| JK134 | *h^+^* *ade6-469 hhp1::natMX4 hhp2::kanMX6* | Table 1 |
| FY183 | *h^+^ pat1-114 rec11-TAP::kanMX6* | Fig. 2 |
| FY184 | *h^+^ pat1-114 hhp1::natMX4 hhp1-as::hphMX4 hhp2::kanMX6 rec11-TAP::kanMX6* | Fig. 2 |
| JG17731 | *h^-^ ade6-3049 pat1-114 rec11-TAP::bleMX6 hhp1::natMX4 hhp1-as(M84G)::hphMX4 hhp2::kanMX6* | Fig. S4 |
| JG17731 | *h^-^ ade6-3049 pat1-114 rec11-TAP::bleMX6 hhp1::natMX4 hhp1-as(M84G)::hphMX4 hhp2::kanMX6* | Fig. S4 |
| JG17732 | *h^+^ pat1-114 ade6-216 rec11-TAP::bleMX6* | Fig. S4 |
| JG17733 | *pat1-114 ade6 rec11::kanMX6 rec11-10A::hphMX4 rec11-10A-TAP::bleMX6 hhp1::natMX4 hhp1-as(M84G)::hphMX4 hhp2::kanMX6* | Fig. S4 |
| JG17735 | *pat1-114 ade6 rec11::kanMX6 rec11-10D::hphMX4 rec11-10D-TAP::bleMX6 hhp1::natMX4 hhp1-as(M84G)::hphMX4 hhp2::kanMX6* | Fig. S4 |
| JG17323 | *h^-^ rec10-203::GFP-kanMX6 pat1-114 ade6-210* | Fig. 3 and S7 |
| JG17411 | *h^+^ rec27-205::GFP-kanMX6 pat1-114 ade6-*(*210* or *3049*) | Fig. 3 and S7 |
| JG17327 | *h^-^ rec10-203::GFP-kanMX6 hhp1::natMX4 hhp1-as(M84G)::hphMX4 hhp2::kanMX6 pat1-114 ade6-3049* | Fig. 3 and S7 |
| JG17326 | *h^-^ rec27-205::GFP-kanMX6 hhp1::natMX4 hhp1-as(M84G)::hphMX4 hhp2::kanMX6 pat1-114 ade6-3049* | Fig. 3 and S7 |
| JG17415 | *h^+^ rec10-203:GFP-kanMX6 pat1-114 rec11::kanMX6 rec11-10A::hphMX4 ade6 ade6-*(*M26* or *210*) | Fig. 3 and S7 |
| JG17413 | *h^+^ rec27-205::GFP-kanMX6 pat1-114 rec11::kanMX6 rec11-10A::hphMX4 ade6 ade6-*(*M26* or *3049*) | Fig. 3 and S7 |
| JG12618 | *h^90^ ade6-216 leu1-32 lys1-131 ura4-D18 cen2(D107)::kan-ura4^+^-lacO his7^+^::lacI-GFP* | Table S1 |
| JG15077 | *h^90^ cen2(D107)::kan-ura4^+^-lacO his7^+^::lacI-GFP hhp1::natMX4 hhp1-as(M84G)::hphMX4 hhp2::kanMX6* | Table S1 |
| JG14848 | *h^-^/h^-^ pat1-114/pat1-114 ade6-210/ade6-216 hhp1-TAP::kanMX6/hhp1-TAP::kanMX6* | Table S4 and Fig. S2 |
| JG14849 | *h^-^/h^-^ pat1-114/pat1-114 ade6-210/ade6-216 hhp2-TAP::kanMX6/hhp2-TAP::kanMX6* | Table S4 and Fig S2 |
| JK135 | *pat1-114/pat1-114 ade6-210/ade6-216 rec11-TAP::kanMX6/rec11-TAP::kanMX6* | Table S4 and Fig. S2 |
| JG15116 | *h^-^/h^-^ pat1-114/pat1-114 ade6-210/ade6-216 hhp2-TAP::kanMX6/hhp2-TAP::kanMX6 hhp1-PK9::hphMX4* | Fig. S3 |
| JG17411 | *h^+^ rec27-205::GFP-kanMX6 pat1-114 ade6 ade6-*(*210* or *3049*) | Fig. S4A |
| JG17413 | *h^+^ rec27-205::GFP-kanMX6 pat1-114 rec11::kanMX6 rec11-10A::hphMX4 ade6-(M26* or *3049*) | Fig. S4A |
| JG17412 | *h^-^ rec27-205::GFP-kanMX6 pat1-114 rec11::kanMX6 rec11-10D:hphMX4 ade6 ade6-(M26* or *3049*) | Fig. S4A |
| JG12069 | *h^90^ rec8::kanMX<<ura4^+^<<Prec8-rec8-RDRD-FLAG<<kanMX lys1^+^<<lacO his7^+^<<Pdis1-GFP-lacI-NLS ade6-216 leu1 ura4-D18* | Fig. S4B |
| JG11218 | *h^+^ rec8-GFP::kanMX ade6-210* | Fig. S5 |
| JG11219 | *h^-^ rec8-GFP::kanMX ade6-216* | Fig. S5 |
| JG17330 | *h^+^ rec11::kanMX6 rec11-10D::hphMX4 rec8-GFP::kanMX* (omns)^b^ | Fig. S5 |
| JG17319 | *h^-^ rec11::kanMX6 rec11-10D::hphMX4 rec8-GFP::kanMX* (omns) | Fig. S5 |
| JG17314 | *h^+^ rec11::kanMX6 rec11-10A::hphMX4 rec8-GFP::kanMX* (omns) | Fig. S5 |
| JG17316 | *h^-^ rec11::kanMX6 rec11-10A::hphMX4 rec8-GFP::kanMX* (omns) | Fig. S5 |
| JG12069 | *h^90^ rec8::kanMX<<ura4^+^<<Prec8-rec8-RDRD-FLAG<<kanMX lys1^+^<<lacO his7^+^<<Pdis1-GFP-LacI-NLS ade6-216 leu1 ura4-D18* | Fig. S6 |
| JG15595 | *h^90^ leu1 ura4 lacO-lys1^+^ GFP-LacI-his7^+^ rec8::natMX4 rec8 RD1-ura4^+^* | Fig. S6 |
| JG17709 | *h^90^ leu1 ura4 lacO-lys1^+^ GFP-LacI-his7^+^ rec8::natMX4 rec8 RD1-ura4^+^ rec11-10A::hphMX4* | Fig. S6 |

*^a^* Sources of alleles other than commonly used auxotrophies and mating type are: *ade6-3049* [4]; *ade6-3061* [1]; *arg3-D4* [21]; *cen2(D107)::kan-ura4^+^-lacO* [5]; *end1-458* [6]; *hhp1::natMX4* [7]; *hhp1-as(M84G)::hphMX4* [7]; *hhp1-TAP::kanMX6,* Materials and Methods; *hhp1-PK9::hphMX4,* Material and Methods; *hhp2::kanMX6,* Materials and Methods; *hhp2-TAP::kanMX6,* Materials and Methods; *his7^+^::lacI-GFP* [5]; *his7^+^::lacI-GFP* [5]*; his7^+^<<Pdis1-GFP-lacI-NLS* [8]; *lys1^+^<<lacO* [8]; *pat1-114* [9]; *rad50S* [10]; *rec8::kanMX<<ura4^+^<<Prec8-rec8-RDRD-FLAG<<kanMX* [11]; *rec8-RD1* [11]; *rec8::kanMX6<<ura4^+^<<Prec8-rec8N12A-GFP::kanMX6* [2]; *rec8::natMX4* [3]; *rec8-176::kanMX6* [12]; *rec8-FLAG-Tspo5::natMX4* [2]; *rec8-7A-FLAG-Tspo5::natMX4* [2]; *rec8-S412A-FLAG-Tspo5::natMX4* [2]; *Prec8-rec8-N12A-S412A-GFP::kanMX6* [2]; *rec8-17AS412A-ura4^+^::kanMX6* [2]; *rec8-GFP::kanMX* [13]; *rec8-ST17A-ura4^+^::natMX4* [2]; *rec10-203-GFP::kanMX6* [14]; *rec11::kanMX6* [15]; *rec11^+^::hphMX4,* Materials and Methods*; rec11-10A::hphMX4,* Materials and Methods*; rec11-10D::hphMX4,* Materials and Methods*; rec11-GFP::kanMX6* [15]*; rec11-TAP::kanMX6,* Materials and Methods; *rec12-152::LEU2* [16]; *rec12-172::I-SceI* [17]; *rec12-201::6His-2FLAG* [18]; *rec27-205-GFP::kanMX6* [19]; *tps16-23* [20]. Sequences of TAP and PK tags are described in [22] and [23], respectively.

^b^ omns: other auxotrophic markers not scored.
